# Triacylglycerol synthesis in microalgae under abiotic stress: an updated integrative review

**DOI:** 10.1093/pcp/pcag059

**Published:** 2026-05-07

**Authors:** Vipul Swarup Bhatnagar, Marta Saldat, Agnieszka Zienkiewicz, Zhi-Yan Du, Krzysztof Zienkiewicz

**Affiliations:** Institute of Advanced Sciences, Centre for Modern Interdisciplinary Technologies, Nicolaus Copernicus University in Toruń, Wileńska 4, 87-100 Toruń, Poland; Institute of Advanced Sciences, Centre for Modern Interdisciplinary Technologies, Nicolaus Copernicus University in Toruń, Wileńska 4, 87-100 Toruń, Poland; Institute of Advanced Sciences, Centre for Modern Interdisciplinary Technologies, Nicolaus Copernicus University in Toruń, Wileńska 4, 87-100 Toruń, Poland; Department of Molecular Biosciences & Bioengineering, University of Hawaii at Manoa, 1955 East-West Road, Honolulu, HI 96822, USA; Institute of Advanced Sciences, Centre for Modern Interdisciplinary Technologies, Nicolaus Copernicus University in Toruń, Wileńska 4, 87-100 Toruń, Poland

**Keywords:** abiotic stress, lipid, lipid droplets, microalgae, triacylglycerol (TAG)

## Abstract

Microalgae exhibit remarkable metabolic plasticity, rapidly redirecting carbon toward triacylglycerol (TAG) accumulation under environmental stress. In this review, we synthesize recent advances in understanding how nutrient limitation, light fluctuations, salinity, temperature extremes, and chemical modulators regulate TAG biosynthesis in diverse microalgal systems. Under stress, TAG accumulation is typically linked to the coordinated transcriptional and metabolic reprogramming of the glycerol-3-phosphate (Kennedy) pathway, with diacylglycerol acyltransferases acting as the pivotal enzymes that complete TAG biosynthesis. However, these responses are highly species- and isoform-specific, reflecting substantial evolutionary divergence. In addition to enzyme-level control, increasing evidence points to a central role for intracellular signaling networks and transcription factors in coordinating these responses. Although upstream regulatory mechanisms differ, their outcomes commonly result in enhanced TAG accumulation and the remodeling of fatty acid pools. Together, these findings support a hierarchical model in which environmental sensing, signal integration, and transcriptional regulation coordinate TAG biosynthesis as an adaptive response to environmental stressors.

## Introduction

Microalgae are unicellular eukaryotic photosynthetic organisms that play a fundamental role in aquatic ecosystems, contributing substantially to global primary production, oxygen evolution, and carbon dioxide fixation (Naselli-Flores and Padisák 2023). Through oxygenic photosynthesis, they efficiently convert solar energy into chemical energy and diverse organic compounds, forming the base of many aquatic food webs. In addition to their ecological importance, microalgae exhibit remarkable metabolic diversity and the capacity to synthesize a wide range of bioactive molecules, including pigments, proteins, carbohydrates, and lipids (Khan et al. 2018, Shitanaka et al. 2026). Among these metabolites, lipids, particularly neutral lipids in the form of triacylglycerol (TAG), have attracted considerable attention. TAG represents a highly reduced, energy-dense molecule that serves as major carbon and energy reserves in diverse types of cells. In many microalgal species, the content of TAG can reach exceptionally high levels, sometimes exceeding 50% of the dry biomass under specific environmental conditions (Xu 2022). Besides their function as storage compounds, TAG molecules are currently recognized as key regulators of cellular homeostasis, contributing to redox balance, buffering excess fatty acids (FAs), protecting membrane integrity, and supporting stress tolerance, similarly to their roles in higher plants and other eukaryotes (Ischebeck et al. 2020, Winichayakul et al. 2025).

The exceptional capacity of microalgae for the fast and efficient redirection of carbon flux toward TAG synthesis is strongly influenced by environmental factors such as nutrient availability, light intensity, salinity, and temperature (Zienkiewicz et al. 2016, Sun et al. 2018a, Li-Beisson et al. 2019, Suparmaniam et al. 2023). Exposure to abiotic stress often triggers rapid metabolic reprogramming in microalgal cells, leading to growth arrest and a shift of cellular resources toward TAG accumulation (Zienkiewicz et al. 2016, 2020). However, these responses are highly variable across taxa, reflecting the exceptional evolutionary, ecological, and physiological diversity of microalgae. Consequently, understanding how TAG biosynthesis is regulated at the molecular, biochemical, and cellular levels in response to environmental stimuli currently remains one of the central challenges in microalgal biology.

The regulatory mechanisms controlling TAG synthesis and accumulation also need to be considered in the context of the remarkable diversity of microalgae, which span a wide range of evolutionary lineages, including green algae (*Chlorophyta*), red algae (*Rhodophyta*), stramenopiles (e.g. diatoms), haptophytes, and dinoflagellates. These groups differ in plastid origin, arising from primary or secondary endosymbiosis, as well as in cellular architecture, membrane organization, and metabolic compartmentalization (Gentil et al. 2017). These differences have important consequences for FAs trafficking, lipid assembly, and the regulation of stress responses. Microalgae also vary widely in their ecology and physiology. They are found in freshwater, marine, hypersaline, polar, and tropical environments, and they use a range of trophic strategies, from strict photoautotrophy to mixotrophy and heterotrophy (Chen and Chen 2006; Perez-Garcia et al. 2011; Bumbak et al. 2011; Morales-Sánchez et al. 2015). Some species are motile and can adjust their position in response to light or other cues, while others remain fully exposed to environmental changes. Together, these evolutionary, ecological, and physiological differences shape how microalgae perceive and respond to stress, and ultimately, how TAG biosynthesis is regulated. For reference, the major species discussed in this review, along with their phylogenetic affiliation, plastid origin, habitat, and trophic traits, are summarized in Supplementary Table S1.

Over the past two decades, advances in the molecular biology of microalgae, including high-quality genome assemblies, transcriptomic datasets, and genetic tools, have enabled detailed studies of their lipid metabolism (Blaby-Haas and Merchant 2019, Schroda and Remacle 2022, Einhaus et al. 2024). Many studies also show that environmental stress triggers highly coordinated changes in the expression and activity of TAG biosynthetic enzymes, shaping both lipid yield and FAs composition. However, these responses are very often species- and strain-specific, highlighting the need for comparative and integrative analyses across diverse microalgal lineages.

Several comprehensive and influential reviews have already summarized diverse aspects of lipid metabolism and stress-induced TAG accumulation in microalgae (Hu et al. 2008, Griffiths and Harrison 2009, Zienkiewicz et al. 2016, Yaakob et al. 2021, Song et al. 2022). However, the field has advanced rapidly in recent years. New insights from genomics, transcriptomics, functional studies of lipid metabolic enzymes, and systems-level analyses have significantly expanded our understanding of TAG biosynthesis and its regulation in microalgae, providing a strong basis for a more integrative synthesis of recent work. Building on these advances, here we review how TAG biosynthesis is regulated in microalgae under diverse abiotic and chemical stress conditions, across multiple genera and evolutionary lineages. We focus primarily on the genes directly involved in TAG formation as well as on the associated metabolic and regulatory networks that govern TAG assembly, storage, and mobilization. This review emphasizes original research published between 2018 and 2025 that includes quantitative analyses of gene expression, enzyme activity, and lipid content and composition, while incorporating key earlier studies where relevant. Rather than using strict exclusion criteria, we take a comparative and integrative approach that evaluates mechanistic, regulatory, and physiological data across diverse species and stress conditions.

We specifically emphasize the mechanistic basis of TAG biosynthesis, the transcriptional control of lipid metabolism, associated changes in lipid profiles, and, finally, upstream processes such as stress perception and intracellular signaling. By integrating findings across diverse species and stress conditions, we identify shared regulatory features as well as lineage-specific differences that shape TAG accumulation. This approach also highlights key gaps that remain unresolved, particularly in linking upstream signaling events with the metabolic flux toward TAG synthesis. Together, these insights provide a clearer framework for understanding TAG biosynthesis in microalgae and provide a foundation for future research and applied strategies.

## Core Pathways of Triacylglycerol Biosynthesis in Microalgae

The biochemical pathways of TAG synthesis in microalgae and higher plants have been extensively characterized over the past decades. Here, we provide a concise overview of the core molecular framework to establish the background for the following sections. The canonical TAG biosynthetic pathway outlined below is primarily derived from functional and genetic studies on the model green alga *Chlamydomonas reinhardtii*, with comparative references to additional microalgal taxa where relevant.

### Carbon flux and fatty acids synthesis in plastids

**Figure 1:**
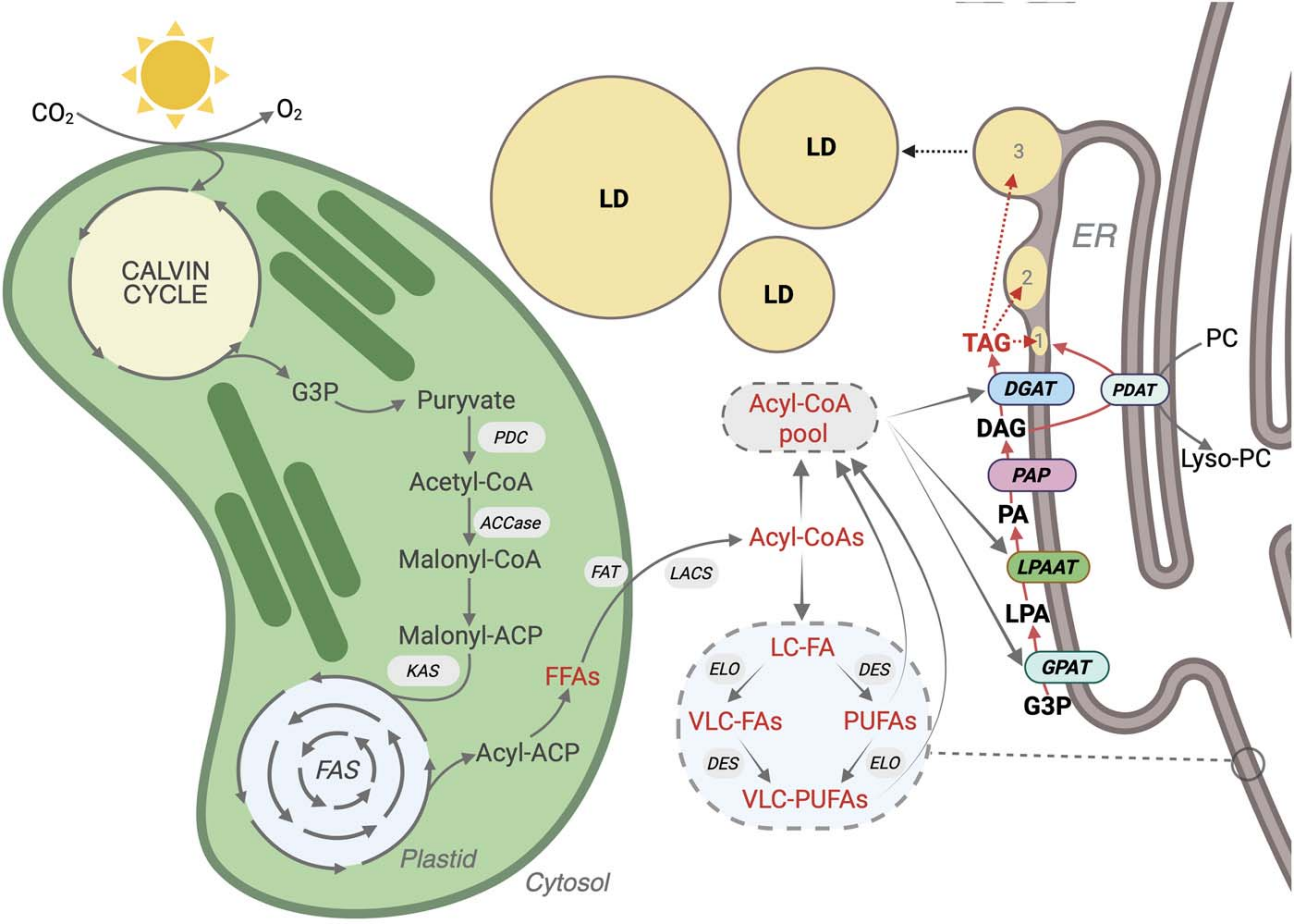
Overview of FAs and TAG synthesis pathways in microalgal cells. FA synthesis occurs in the plastids, where malonyl-CoA serves as a substrate. Within the FAS complex, the FA, bound to the ACP protein, is transferred through various subunits of the complex. A series of condensation and elongation steps catalyzed by FAS results in the formation of acyl-ACPs, which are subsequently used for the synthesis of diverse lipid classes. Once exported to the cytosol, acyl-ACPs are hydrolyzed by thioesterases (FAT), contributing to the acyl-CoA pool in the cytosol. Acyl-CoAs can also be elongated and desaturated in the ER to form VLC-PUFAs, further enriching the cytosolic acyl-CoA pool. The primary pathway for TAG synthesis occurs in the ER and is catalyzed by several conserved enzymes located at the ER membrane. In the first step, G3P undergoes esterification with acyl-CoA to form LPA, catalyzed by GPAT. LPA then serves as a substrate for a second acyltransferase, LPAAT, which converts it into PA. Next, PAP dephosphorylates PA to form DAG, which acts as the direct precursor for TAG synthesis. The final step in TAG biosynthesis is catalyzed by DGAT, marking the committed step in TAG formation. An alternative pathway where PDAT catalyzes a reaction in which PC donates an acyl group to DAG to form TAG has also been described for several microalgae. Newly formed TAG is gradually accumulated in LDs (1→3), which initially form at the ER membrane and later bud off into the cytosol. ACCase, acetyl-CoA carboxylase; ACP, acyl carrier protein; CoA, coenzyme A; DAG, diacylglycerol; DGAT, acyl-CoA:diacylglycerol acyl transferase; FAS, fatty acid synthase complex; FAT, fatty acyl-ACP thioesterase; FFAs, free fatty acids; KAS, 3-ketoacyl-ACP synthase; G3P, glycerol 3 phosphate; GPAT, acyl-CoA:glycerol-3-phosphate acyl transferase; LACS, long chain acyl-CoA syn-thetase; LPA, lysophosphatidic acid; LPAAT, acyl-CoA:lysophosphatidic acid acyl transferase; LPCAT, acyl-CoA:lysophosphatidylcholine acyltransferase; PA, phosphatidic acid; PAP, phosphatidic acid phosphatase; PUFA, polyunsaturated fatty acid; TAG, triacylglycerol; VLC, very long chain. Created in BioRender. Zienkiewicz, K. (2026). https://BioRender.com/cmf9u7x.

As in plants, *de novo* FAs synthesis in photosynthetic microalgae occurs predominantly in the plastid and is tightly coupled to photosynthetic carbon assimilation. The initial steps of lipid biosynthesis rely on intermediates and reducing power generated through the Calvin cycle (Fig. 1). Within the plastid stroma, ribulose-1,5-bisphosphate carboxylase/oxygenase (Rubisco) catalyzes the CO₂ fixation into 3-phosphoglycerate (3-PGA), which is then reduced to glyceraldehyde-3-phosphate (G3P) using the adenosine triphosphate (ATP) and nicotinamide adenine dinucleotide phosphate (NADPH) produced during the light reactions of photosynthesis (Gurrieri et al. 2021). G3P holds a central metabolic position, as it can be redirected toward pyruvate formation and ultimately converted into acetyl-coenzyme A (CoA), the primary building block for the biosynthesis of FAs in plastids (Li-Beisson et al. 2019). FAs synthesis is initiated by the carboxylation of acetyl-CoA to malonyl-CoA, catalyzed by acetyl-CoA carboxylase (ACCase). Malonyl-CoA then enters the FA synthase (FAS) complex, where iterative elongation cycles generate predominantly C16:0 and C18:0 acyl chains bound to the acyl carrier protein (ACP). Each elongation step requires ATP and NADPH, highlighting the central role of photosynthetic energy metabolism in FAs production (Li-Beisson et al. 2019, Chen and Wang 2021). Chain termination is mediated by fatty acyl-ACP thioesterases (FATs), which release free FAs (FFAs) from ACP (Kalinger et al. 2020) (Fig. 1).

These plastid-derived FAs can then be further modified in other cellular compartments. In this context, it is important to distinguish between FAs synthesized *de novo* in plastids and those subsequently processed in the endoplasmic reticulum (ER). Consistent with this, plastidial synthesis primarily produces C16 and C18 acyl chains, whereas elongation and desaturation in the ER generate long-chain and very-long-chain polyunsaturated FAs (LC-PUFAs and VLC-PUFAs, respectively), increasing both chain length and the degree of unsaturation (Jesionowska et al. 2023). As a result, although microalgal glycerolipids may contain FAs ranging from C14 to C24, the initial plastid-derived products are mainly C16 and C18 species, with additional structural diversity arising from ER-based modifications (Maltsev and Maltseva 2021).

Following their release from ACP, FFAs are activated to acyl-CoA esters by long-chain acyl-CoA synthetases (LACS) and exported from the plastid. Although the plastid-to-ER transport of FAs has been characterized at a general level in green algae, largely based on studies in the model *C. reinhardtii*, the exact trafficking mechanisms remain incompletely resolved and may differ in lineages harboring secondary plastids (Peter et al. 2022). In general, acyl-CoAs are transferred to the cytosol and subsequently to the ER, where they serve as substrates for glycerolipid and TAG assembly (Fig. 1).

### Endoplasmic reticulum-based triacylglycerol assembly

In most eukaryotic cells, including microalgae, the principal route of TAG synthesis is the G3P pathway, commonly referred to as the Kennedy pathway (Weiss et al. 1960). This pathway operates predominantly at the ER membrane and utilizes fatty acyl-CoAs produced in plastids and further modified in the ER.

TAG assembly occurs through a sequential series of acylation and dephosphorylation reactions. First, sn-glycerol-3-phosphate acyltransferase (GPAT) catalyzes the acylation of G3P to form lysophosphatidic acid (LPA). LPA is then acylated by LPA acyltransferase (LPAAT) to generate phosphatidic acid (PA). Subsequent dephosphorylation of PA by PA phosphatase (PAP) produces sn-1,2-diacylglycerol (DAG), the immediate precursor of TAG. In the final and committed step of the pathway, DAG is acylated by DAG acyltransferase (DGAT), yielding TAG (Li-Beisson et al. 2019, Chen and Wang 2021). DGATs are widely considered the rate-limiting enzymes of TAG biosynthesis and thus, represent one of the key players in cellular lipid metabolism. Two major membrane-bound DGAT types, DGAT1 and DGAT2, have been characterized in eukaryotes, including microalgae (Maraschin et al. 2019). In addition, a soluble DGAT3 isoform lacking transmembrane domains has been identified in several plant and microalgal species and may contribute to TAG accumulation under specific physiological conditions (Carro et al. 2022, Trenz et al. 2022).

In contrast to plants, yeast, and animals, which typically encode only one or a few DGAT-encoding genes, many microalgal genomes contain multiple *DGAT* isoforms, particularly those encoding DGATs of type 2 (often referred to as DGTTs to distinguish them from type 1 DGATs) (Zienkiewicz et al. 2016). Comparative genomic analyses further reveal substantial variation in their copy number across microalgal lineages and even among strains of the same species. For example, *C. reinhardtii* encodes five DGTT isoforms, whereas species of the genus *Nannochloropsis* possess 11–12 DGTT-encoding genes in their genomes (Vieler et al. 2012, Hung et al. 2013, Li et al. 2014). This expansion is generally interpreted as reflecting the functional diversification of *DGAT* isoforms, including expression profiles, differences in substrate specificity, or subcellular localization (Zienkiewicz *et al.* 2016, 2018, 2020, Mao et al. 2019, Ma et al. 2022). A summary of *DGAT* isoform copy numbers across representative microalgal taxa, including their classification by type, is provided in Supplementary Table S2.

In addition to DGAT-mediated TAG synthesis, an alternative pathway involves phospholipid:diacylglycerol acyltransferase (PDAT), which transfers an acyl group from phosphatidylcholine (PC) to DAG, generating TAG independently of acyl-CoA (Sah et al. 2024). *PDAT* orthologs have been identified in several microalgae, including *C. reinhardtii* (Yoon et al. 2012) and *Nannochloropsis oceanica* (Yang et al. 2022a), indicating that acyl editing and membrane lipid remodeling contribute to TAG formation.

### Lipid droplet biogenesis and triacylglycerol storage

Newly synthesized TAG molecules are sequestered into specialized organelles known as lipid droplets (LDs), which are ubiquitous in eukaryotic cells (Ischebeck et al. 2020, Bosch and Pol 2022). In the canonical model, TAG accumulates between the two leaflets of the ER membrane, forming lens-like structures that progressively enlarge and bud toward the cytosol, ultimately generating spherical droplets surrounded by a phospholipid monolayer derived from the ER (Renne et al. 2020). During this process, TAGs accumulate within the droplet core, while the surrounding phospholipid monolayer incorporates specific proteins that regulate LD biogenesis, structural stability, and interactions with other cellular compartments (Ischebeck et al. 2020, Zienkiewicz and Zienkiewicz 2020, Guzha et al. 2023).

As in other eukaryotic cells, LDs formation in microalgae is tightly linked to TAG biosynthesis and represents a regulated and spatially organized process rather than a passive consequence of TAG overproduction. Based largely on models developed in other eukaryotic systems, LDs are thought to originate at specialized ER subdomains enriched in lipid metabolic enzymes and structural scaffold proteins (Nettebrock and Bohnert 2020, Kumari et al. 2023). Proteins of the seipin family play a central role in this process by facilitating TAG nucleation and controlling size and morphology of LDs (Renne et al. 2020, Klug et al. 2024). Recent work in the model diatom *Phaeodactylum tricornutum* has provided the first functional characterization of microalgal seipin, revealing both conserved and lineage-specific features of LDs biogenesis (Le Moigne et al. 2025). Additional LD-associated proteins mediate lipid trafficking, droplet fusion or fragmentation, and interactions with other organelles, including plastids, mitochondria, and peroxisomes (Ischebeck et al. 2020, Bosch and Pol 2022).

LDs biogenesis is strongly induced under environmental stress conditions that promote TAG accumulation. Abiotic stresses such as nutrient limitation, high light intensity, salinity, and temperature fluctuations trigger extensive metabolic reprogramming and redirect carbon flux toward TAG synthesis, leading to LD formation (Zienkiewicz et al. 2016, 2020, Bouchnak et al. 2023). Under these conditions, LDs can accumulate to high abundance and large size within the cytoplasm, as observed in numerous microalgae.

Importantly, the current models no longer view LDs as simple storage sites for TAG but rather as dynamic organelles that play active roles in cellular metabolism, including responses to stress. For example, LDs contribute to lipid homeostasis by facilitating the incorporation of FFAs released during stress-induced membrane remodeling into TAG, thus preventing the accumulation of cytotoxic intermediates (Ischebeck et al. 2020, Bouchnak et al. 2023, Obaseki et al. 2024). In a broader context, LDs are now recognized as central components of lipid metabolism in eukaryotic cells, linking lipid synthesis, remodeling, and turnover processes (Ischebeck et al. 2020; Bosch and Pol 2022). To perform these roles, LDs rely on a diverse and dynamic protein composition. Identified LD-associated proteins include lipid metabolic enzymes, structural components involved in droplet stability and organization, and regulatory factors linked to stress responses and cellular signaling (Ischebeck et al. 2020, Bouchnak et al. 2023, Zhao et al. 2025). In microalgae, LDs contain a diverse and dynamic protein complement that varies across species and physiological conditions (Zienkiewicz and Zienkiewicz 2020, Li-Beisson et al. 2021).

Given their central role in lipid metabolism and TAG storage, LDs are also of considerable biotechnological interest in microalgae. Their capacity to accumulate energy-dense neutral lipids makes them one of the key targets for the production of biofuels, nutraceuticals, and other high-value lipid-derived compounds (Bouchnak et al. 2023, Xu et al. 2023).

### Plastid contributions to triacylglycerol synthesis

While TAG synthesis in microalgae is primarily associated with the ER *via* the Kennedy pathway described above, recent studies show that plastids can also contribute to TAG formation, either directly or indirectly (Li-Beisson et al. 2019). This contribution becomes particularly relevant under stress conditions that promote lipid accumulation and are accompanied by extensive remodeling of plastid membranes.

Thus, in photosynthetic eukaryotes, glycerolipid metabolism may involve two interconnected pathways traditionally referred to as the prokaryotic (plastidial) and eukaryotic (ER-based) routes of lipid synthesis (Li-Beisson et al. 2013). In both pathways, G3P undergoes sequential acylation to LPA, PA, and ultimately DAG, a central intermediate in glycerolipid metabolism. In the plastid-localized (prokaryotic) pathway however, DAG primarily serves as a precursor for membrane glycerolipids such as monogalactosyldiacylglycerol (MGDG) and digalactosyldiacylglycerol (DGDG). In contrast, DAG generated at the ER through the eukaryotic pathway is typically directed toward TAG synthesis, but it can also serve as a precursor for membrane glycerolipids, including phospholipids and, in some microalgae, betaine lipids (Li-Beisson et al. 2019, Salomon et al. 2025). These two pathways can be distinguished by their FA composition, as plastid-derived lipids often contain 16-carbon FAs at the position, whereas ER-derived lipids more frequently contain 18-carbon acyl chains at the same position, reflecting, in part, the substrate specificity of plastidial and ER-localized acyltransferases (Li-Beisson et al. 2013, Li-Beisson et al. 2019). These differences also arise from the plastidial origin of FA synthesis, which supplies acyl substrates to both pathways. However, this distinction is best established in model systems such as *Arabidopsis thaliana* and may not be universally applicable to all microalgae, particularly those with secondary plastids or more complex lipid metabolic networks.

In microalgae, at least few studies provide direct evidence for plastid involvement in TAG metabolism. For example, early studies of Fan et al. (2011) in *C. reinhardtii*, showed that TAG can be synthesized using chloroplast-derived DAG and subsequently stored in LDs located both in the cytosol and within the chloroplast. More recently, plastid-associated localization of TAG biosynthetic enzymes has also been reported in *P. tricornutum*, where specific DGAT isoforms have been detected within plastids or at plastid envelope membranes, indicating that part of TAG assembly may occur in plastid-associated compartments (Zhang et al. 2021, Huang et al. 2024).

## Effects of Abiotic Stressors on Triacylglycerol Biosynthesis

As briefly noted above, environmental fluctuations have long been known to strongly influence carbon allocation and lipid metabolism in microalgae, often redirecting carbon flux toward TAG accumulation (Zienkiewicz et al. 2016, Li-Beisson et al. 2019). Even more than multicellular plants, microalgae are directly exposed to rapidly changing conditions, including changes in nutrient availability, light intensity, salinity, temperature, and other environmental factors. To handle these fluctuations, they rely on complex and flexible regulatory networks that coordinate responses across multiple cellular processes and enable rapid metabolic reprogramming. As a result, carbon metabolism is often redirected to support acetyl-CoA production and reducing power, while key genes involved in TAG synthesis and lipid remodeling are differentially expressed. At the same time, membrane lipids are remodeled or degraded, supplying substrates for TAG biosynthesis and LD formation. These adjustments are often accompanied by shifts in FA composition, including changes in chain length and the degree of unsaturation (see below). However, in some species, increased lipid accumulation may reflect a redistribution within existing lipid metabolic pathways or reduced cellular growth rather than a net increase in carbon flux toward lipid synthesis.

As will be discussed throughout the following sections, the regulatory mechanisms behind these responses very often vary widely between microalgal taxa. Factors such as evolutionary lineage, plastid architecture, trophic mode, ecological origin, and experimental conditions all shape how individual species perceive and respond to stress. In addition, similar stress categories are often applied using different experimental setups, including partial versus complete nutrient limitation or transient versus prolonged exposure. This variability can strongly affect gene expression patterns and lipid outcomes, making direct comparisons between studies challenging.

In the sections below, we review recent studies on how major abiotic stressors influence TAG biosynthesis across diverse microalgal species. For each stress category, we integrate reported transcriptional responses of the Kennedy pathway enzymes and associated regulators with changes in lipid composition, highlighting both shared regulatory patterns and lineage-specific differences. This comparative overview aims to place these individual findings into a broader context while acknowledging the diversity of microalgal stress responses.

### Nutrient stress

Nutrient limitation is one of the most well-established triggers of TAG accumulation in microalgae. Among essential macronutrients, nitrogen (N) and phosphorus (P) play central roles in growth by supporting such essential processes like protein synthesis, nucleic acid production, and membrane biogenesis. When these nutrients become limiting, microalgal cells reduce cell division and the synthesis of N- and P-containing macromolecules, while increasing carbon storage in the form of TAGs (Zienkiewicz et al. 2020, Maltsev et al. 2023). This dramatic shift reflects a rapid and extensive reorganization of cellular metabolism, redirecting carbon away from growth and toward energy storage, which supports survival under nutrient stress and recovery after nutrient resupply.

Such extensive metabolic reorganization is often reflected in pronounced changes in the expression profiles of genes governing diverse aspects of cellular lipid metabolism, including those of the Kennedy pathway (Zienkiewicz et al. 2016). However, as discussed below and summarized in Table 1, the extent and nature of these changes vary widely among microalgal species, likely reflecting differences in their metabolic organization, ecological adaptations, and cultivation conditions. Differences in experimental design should also be considered, as the studies reviewed here range from complete nutrient deprivation to partial limitation, as detailed in Table 1. These approaches are applied under different light regimes, carbon availability, and growth conditions, all of which can influence the observed transcriptional responses.

**Table 1:** Summary of recent studies reporting transcriptional responses of Kennedy pathway genes and major lipid-related changes in microalgae under nutrient stress.

Species	Clade	Ecology/Trophic mode	Stress type	Gene response (Kennedy pathway)	Lipid outcome	FA profile changes	Reference
*Amphora coffeaeformis*	Diatom	Marine/Photoautotroph	N starvation	*GPAT* ▲; *LPAAT* ▲	Neutral lipids ↑ 2.4×; TAG ↑ to 25%; TL ↑ to 45%	SFA ↑; unsaturated FA ↓	Cui et al. (2023)
*Aurantiochytrium* sp*.* T66	Thraustochytrid	Marine/Heterotroph	N limitation	*DGAT1* ▲; *DGAT2* ▼	Lipids ↑ (~55%)	C14:0, C18:1 ↑; DHA ↓ slightly	Heggeset et al. (2019)
*Chaetoceros muelleri*	Diatom	Marine/Photoautotroph	N starvation	*GPAT* ▲; *DGAT2* ▲	TAG ↑	—	de Jesús-Campos et al. (2024)
*Chlamydomonas reinhardtii* (mutant with disrupted *CrbZip2*)	Green algae	Freshwater/Photoautotroph	N starvation	*DGTT3* ▲; *DGTT4* ▲	TAG accumulation impaired	—	Bai et al. (2021)
*Chlorella sorokiniana*	Green algae	Freshwater/Photoautotroph	P limitation	*DGAT* ▼; *LPAAT* ▼	Lipids ↑ then ↓	—	Jin et al. (2021)
*Chlorella sorokiniana*	Green algae	Freshwater/Photoautotroph	N deprivation combined with H_2_O_2_ treatment	*DGAT1* ▲	Lipids ↑	MUFA ↑; PUFA ↓	Liufu et al. (2023)
*Chlorella vulgaris*	Green algae	Freshwater/Photoautotroph	N and P limitation + CO₂ supplementation	*DGAT* isoforms ▼	Lipids ↑ 32%	C16:0, C18:1 ↑	Kumari et al. (2021)
*Chlorella zofingiensis*	Green algae	Freshwater/Photoautotroph	N/P/S stress	*DGAT1a, DGAT2a–f* ▲; *DGAT2g*	TAG ↑	C18:1 ↑; C18:0 ↓	Mao et al. (2018)
*Chlorella zofingiensis*	Green algae	Freshwater/Photoautotroph	N starvation	*DGAT1A* ▲; *DGTT5* ▲	TAG ↑	MUFA ↑; PUFA ↓	Mao et al. (2019)
*Chromochloris zofingiensis*	Green algae	Freshwater/Photoautotroph	S starvation	*PAP3* ▲; *DGATs* ▲; *GPAT*; *LPAAT*; *PAP1/2* ▼	TAG ↑	C16–C18 enriched	Mao et al. (2020b)
*Coccomyxa* sp.	Green algae	Freshwater/Photoautotroph	N starvation	*DGAT2d* ▲	TAG ↑	C16/C18 ↓ ratio	Oyama et al. (2022)
*Dunaliella tertiolecta*	Green algae	Marine/Photoautotroph	N starvation	*GPAT* ▼; *LPAAT* ▲; *DGAT*	Neutral lipids ↑ 6×	C16:0 ↑; PUFA ↓	Tan et al. (2016)
*Dunaliella salina*	Green algae	Marine/Photoautotroph	N limitation (high C/N ratio)	*DGAT2* ▲	TAG ↑	—	Jia et al. (2023)
*Ettlia oleoabundans*	Green algae	Freshwater/Photoautotroph	N starvation	*GPAT* ▲; *PAP* ▲; *LPAAT* ▲; *DGAT* ▲	TAG ↑	C18:1 ↑; PUFA ↓	Sturme et al. (2018)
*Graesiella emersonii*	Green algae	Freshwater/Photoautotroph	N limitation + hormones	*GPAT* ▲	Lipids ↑	SFA ↑; PUFA ↓	Mandal et al. (2020)
*Haematococcus pluvialis*	Green algae	Freshwater/Photoautotroph	N starvation	*GPAT* ▲; *PDAT ▲*	Lipids ↑	—	Y. Zhao et al. (2020b)
*Karenia mikimotoi*	Dinoflagellate	Marine/Photoautotroph	N starvation	*GPAT1* ▲; *GPAT2* ▲	—	—	Shi et al. (2021)
*Nannochloropsis oceanica*	Eustigmatophyte	Marine/Photoautotroph	N starvation	*GPAT* ▲; *LPAAT* ▲	Lipids ↑	C16:0 ↑; PUFA ↓	Ouyang et al. (2024)
*Nannochloropsis oceanica*	Eustigmatophyte	Marine/Photoautotroph	N starvation	*DGTT1–6* ▲; *DGAT1* ▼	TAG ↑	C18:1 ↑; EPA ↓	Zienkiewicz et al. (2020)
*Nannochloropsis gaditana*	Eustigmatophyte	Marine/Photoautotroph	N + P stress	DGAT ▲ (protein)	TAG ↑	C16 enriched	Hulatt et al. (2020)
*Nannochloropsis gaditana*	Eustigmatophyte	Marine/Photoautotroph	N starvation	*DGAT2a/b/g/e* ▲; *DGAT2c/k* ▼	TAG ↑	C18:1 ↑	Janssen et al. (2020)
*Nannochloropsis salina*	Eustigmatophyte	Marine/Photoautotroph	N starvation	*DGTT5* ▲	TFA ↑	C16:0 ↑; PUFA ↓	S.W. Jeong et al. (2017b)
*Neochloris oleoabundans*	Green algae	Freshwater/Photoautotroph	N starvation	*GPAT* ▲; *PAP* ▲; *LPAAT* ▼; *DGAT* ▼	TAG ↑	C18:1 ↑	de Jaeger et al. (2018)
*Phaeodactylum tricornutum* (two different ecotypes)	Diatom	Marine/Photoautotroph	N starvation	*GPAT1* ▲; *LPAAT5* ▲; *DGAT1* ▲	Lipids ↑	C16:0 ↑; EPA ↑	Murison et al. (2023)
*Phaeodactylum tricornutum*	Diatom	Marine/Photoautotroph	N starvation	*DGAT* ▲; *LPAAT* ▼	TAG ↑	—	Scarsini et al. (2022)
*Phaeodactylum tricornutum*	Diatom	Marine/Photoautotroph	N starvation	*DGAT1* ▲; *DGAT2b* ▲ other *DGAT2s*	TAG ↑	C16:1 ↑	Remmers et al. (2017)
*Phaeodactylum tricornutum*	Diatom	Marine/Photoautotroph	N and P stress	*DGATs* ▲; *PDAT* ▲	TAG ↑	—	Huang et al. (2019)
*Scenedesmus acutus*	Green algae	Freshwater/Photoautotroph	N starvation	*GPAT*; *DGAT*	TAG ↑	—	Sirikhachornkit et al. (2018)
*Scenedesmus* sp.	Green algae	Freshwater/Photoautotroph	P limitation	*DGAT1* ▲	Lipids ↑	C16:0 ↑	Yang et al. (2018)
*Skeletonema marinoi*	Diatom	Marine/Photoautotroph	N and P stress	*GPAT* ▲then▼; *DGAT* ▲	TAG ↑	—	M. Zhang et al. 2020a, Y. Zhang et al. 2020a
*Xanthonema hormidioides*	Xanthophyte	Freshwater/Photoautotroph	N stress + temperature variations	*DGAT* ▲; *PDAT* ▲; *GPAT* ▲	Lipids ↑	C16:1 ↑	Gao et al. (2023)
*Schizochytrium* sp.	Thraustochytrid	Marine/Heterotroph	B12 deficiency	*GPAT* ▲; *LPAAT* ▲; *PAP* ▲; *DGAT1* and *DGTT1* ▲; *LPAAT2* and *LPAAT*3 ▼; *DGAT2*▼, *PDAT*▼	Lipids ↑	EPA ↑	Yang et al. (2024)

Species are grouped by major phylogenetic lineages, with ecological origin and trophic mode indicated. Gene expression changes are indicated by arrowheads (▲, upregulation; ▼, downregulation; no symbol, no changes in expression). Lipid-related changes are shown using standard arrows (↑, increase; ↓, decrease). Differences in experimental conditions (e.g. nutrient limitation versus starvation or combined stresses) are also indicated and should be considered when comparing studies.

The following sections summarize recent studies on the effects of N and P limitation on TAG accumulation across diverse microalgae. To keep the presentation clear and consistent, we first review changes in the expression of Kennedy pathway genes across diverse species, typically following the pathway order from *GPAT* to *LPAAT, PAP*, and *DGAT*, and then we describe the corresponding changes in lipid content and composition. Additional details are provided in Table 1.

#### Nitrogen limitation

With respect to the Kennedy pathway genes, numerous studies have shown that changes in the availability of this N deprivation can strongly affect their expression, a trend further supported by recent data (Table 1). For instance, the N-responsive regulation of GPAT-encoding genes has indeed been reported in numerous microalgae. In the model diatom *P. tricornutum, PtGPAT* transcript abundance increased approximately 2.3-fold under N starvation (Murison et al. 2023), coinciding with an increase in TAG content. Isoform-specific regulation has also been reported in this species, where *PtGPAT2* was upregulated while *PtGPAT1* was downregulated during N deprivation, correlating with enhanced neutral lipid accumulation (Scarsini et al. 2022). Upregulation of GPAT-encoding genes under N limitation has also been documented in several other microalgae, including green algae such as *Haematococcus pluvialis* (Y. Zhao et al. 2020b), *Nannochloropsis gaditana* (Janssen et al. 2020), or *N. oceanica* (Ouyang et al. 2024), as well as diatoms and dinoflagellates such as *Skeletonema marinoi* (M. Zhang et al. 2020a, Y. Zhang et al. 2020a), *Amphora coffeaeformis* (Cui et al. 2023), *Karenia mikimotoi* (Shi et al. 2021), and *Chaetoceros muelleri* (de Jesús-Campos et al. 2024) (Table 1). Nevertheless, the N-induced regulation of *GPAT*s is not universal. In the green alga *Scenedesmus acutus, GPAT* transcript levels remained largely unchanged under N deprivation (Sirikhachornkit et al. 2018), whereas several *Dunaliella* species exhibited the downregulation of *GPAT*s under similar conditions (Tan et al. 2016, Lin et al. 2018).

As shown in Table 1, N limitation also affects the expression of other acyltransferases in the Kennedy pathway. The expression of *LPAAT* responds to N stress in a species-dependent manner. For example, a slight downregulation of *LPAAT* transcripts has been reported in *C. reinhardtii* (Chen et al. 2023) and the red alga *Porphyridium cruentum* (Wei et al. 2021) under N-limited conditions. In contrast, studies in *N. gaditana* have reported either unchanged *LPAAT* expression (Hulatt et al. 2020) or isoform-specific regulation, with two isoforms upregulated and one downregulated (Janssen et al. 2020), accompanied by increases in oleic acid (C18:1) and EPA in the TAG profile.

PAP-encoding genes also frequently respond to N limitation, although their regulation appears less consistent. Upregulation has been reported in model species such as *C. reinhardtii* (Deng et al. 2013) and *N. oceanica* (Zienkiewicz et al. 2020). However, more recent work in the diatom *P. tricornutum* points to a more complex pattern. In nitrogen-deprived cultures, *PtPAP* expression decreased by 4.2-fold despite substantial TAG accumulation (Murison et al. 2023). Isoform-specific responses have also been observed in this species, with *PtPAP2* upregulated and *PtPAP3* downregulated under N limitation, coinciding with enhanced neutral lipid accumulation relative to N-replete conditions (Scarsini et al. 2022).

Many previous and recent studies have strongly expanded the available datasets on microalgal *DGAT*s expression, directly linking these enzymes to TAG formation and complementing earlier findings (Zienkiewicz et al. 2016, Table 1). As already mentioned, *DGAT* genes are frequently expanded in microalgal genomes, and their expression often responds strongly to N deprivation. For example, in *P. tricornutum*, early studies identified *PtDGAT1* and one *DGAT2* isoform as strongly upregulated under N limitation (Guihéneuf et al. 2011, Gong et al. 2013). However, other transcriptomic analyses in this species reported minimal changes in *PtDGAT* expression despite substantial TAG accumulation, suggesting that post-transcriptional regulation, enzyme activity, or substrate availability may also control carbon flux through the pathway (Remmers et al. 2017). A striking example of *DGAT* diversification under N limiting conditions occurs in the genus *Nannochloropsis*. In *N. oceanica*, numerous DGAT-encoding genes, especially *DGTT*s are differentially expressed during N starvation, reflecting the complex evolution and possible specialization of this gene family. For instance, several *DGTT*s genes encoded by the genome of *N. oceanica* CCMP1779 were strongly induced during N starvation and subsequently downregulated upon N resupply, correlating with TAG accumulation and subsequent mobilization (Zienkiewicz et al. 2020). Comparable strain-specific differences have also been reported in *N. gaditana*, where *DGTT* isoforms showed divergent transcriptional responses to N stress depending on the strain background and cultivation conditions (Hulatt et al. 2020, Janssen et al. 2020). For example, in *N. gaditana*, only a single DGAT protein was detected at the proteomic level (Hulatt et al. 2020), despite the presence and transcription of multiple *DGAT* genes, whereas in the *N. gaditana* CCFM-01 strain, four *DGTT* isoforms were significantly upregulated and two were downregulated under N stress (Janssen et al. 2020).

Besides the model microalgae, numerous other species exhibit strong *DGAT* induction during N limitation (Table 1). For example, in *Coccomyxa* sp. it was shown that among seven *DGTT* isoforms encoded by its genome, only one (*DGAT2d*) was strongly upregulated under N stress, with transcript levels increasing up to 50-fold after 10 days of cultivation (Oyama et al. 2022). Similar responses have been reported in the DHA-producing thraustochytrid *Aurantiochytrium* sp. T66, where *DGAT*s of both types were upregulated under N deprivation, accompanied by an increase in total lipid content (Heggeset et al. 2019). Additional examples include *Chlorella kessleri, Dunaliella salina, Xanthonema hormidioides*, and *Graesiella emersonii*, all of which exhibit enhanced *DGAT* expression in response to N stress (Otaki et al. 2019, Mandal and Chaurasia 2021, Gao et al. 2023, Jia et al. 2023).

Together with earlier findings, recent studies reinforce the view that, despite substantial variability at the transcriptional level, N stress consistently induces pronounced and conserved changes in lipid content and composition in microalgal cells, as summarized in Table 1. In most species examined, N limitation leads to substantial increases in total lipid and TAG content, often ranging from ~ 1.5- to more than 3-fold, depending on the species and cultivation conditions. For example, in *N. salina*, N deprivation resulted in a 2-fold increase in total FAs (TFAs), accompanied by the enrichment of saturated and monounsaturated FAs (S.W. Jeong et al. 2017b). In *Coccomyxa* sp., prolonged N stress triggered strong TAG accumulation together with marked changes in acyl chain distribution (Oyama et al. 2022). Similarly, in the abovementioned red microalga *P. cruentum* and the green oleaginous *Ettlia oleoabundans* (*Neochloris oleoabundans*), N limitation promotes lipid accumulation accompanied by a shift in FAs composition toward more saturated species (Sturme et al. 2018, Wei et al. 2021). This pattern is rather consistent across diverse microalgal species, where TAG accumulation under N stress is generally associated with reduced levels of polyunsaturated FAs (PUFAs) and increased proportions of saturated and monounsaturated FAs (SFAs and MUFAs), particularly C16:0, C16:1, and C18:1 (S.W. Jeong et al. 2017b; Oyama et al. 2022; Wei et al. 2021; Sturme et al. 2018; Murison et al. 2023). These changes in the FA pool toward less unsaturated acyl chains likely reflect both membrane lipid degradation and the preferential incorporation of simpler acyl species into TAG. Despite species-specific differences in transcriptional responses, this broadly conserved pattern of lipid remodeling suggests a shared adaptive strategy that favors the accumulation of chemically stable energy storage molecules under nitrogen limitation.

Overall, the contrasting transcriptional responses of the Kennedy pathway genes highlight the diversity of regulatory strategies governing lipid biosynthesis across microalgal lineages. However, these changes alone do not fully explain the extent of TAG accumulation observed under N limitation. Rather, lipid storage under stress reflects integrated metabolic reprogramming that involves coordinated carbon reallocation, membrane lipid remodeling, and regulatory processes besides transcription, including post-transcriptional and enzymatic control.

#### Phosphorus limitation

Similar to N deprivation, P limitation strongly affects essential processes such as cell proliferation and membrane biogenesis. Consequently, P-deprived microalgae undergo extensive metabolic reprogramming, characterized by the suppression of P-demanding processes and the redirection of carbon flux toward TAG.

Consistent with earlier reports, and further supported by more recent data, these metabolic changes are accompanied by coordinated changes in the expression of multiple specific genes, including those involved in TAG biosynthesis, although these responses vary among species and experimental conditions (Table 1). This regulatory effect is particularly evident, e.g. in diatoms. In *S. marinoi*, phosphate limitation resulted in the upregulation of both *GPAT* and *DGAT* (M. Zhang et al. 2020a, Y. Zhang et al. 2020a). Similarly, the model diatom *P. tricornutum* responds to Pstress by upregulating *DGAT* and *PDAT* isoforms, indicating that both acyl-CoA–dependent and phospholipid-dependent pathways may contribute to TAG formation under these conditions (Huang et al. 2019).

Besides diatoms, recent studies summarized in Table 1 highlight that P-dependent regulation of TAG biosynthesis occurs also in green microalgae, although with greater variability in transcriptional responses. For example, in *Scenedesmus* sp., P limitation significantly increased *DGAT1* expression and promoted carbon reallocation toward storage lipid biosynthesis, accompanied by reduced carbohydrate accumulation (Yang et al. 2018). In contrast, *C. vulgaris* showed the downregulation of several DGAT-encoding genes when P limitation was combined with elevated CO₂ and N deprivation (Kumari et al. 2021).

As in the case of N limitation, P deprivation is also commonly associated with extensive membrane lipid remodeling and increased TAG accumulation in microalgae. This characteristic response often involves the replacement of membrane phospholipids with non-phosphorus membrane lipids, including glycolipids such as DGDG and sulfoquinovosyldiacylglycerol, as well as betaine lipids in some species, allowing cells to conserve phosphorus while maintaining membrane function (Khozin-Goldberg and Cohen 2006, Martin et al. 2011, Iwai et al. 2014, Abida et al. 2015, Mühlroth et al. 2017). This remodeling process releases FAs from membrane lipids, which can then be redirected into TAG synthesis. Consistent with earlier observations, recent studies support this mechanism, showing that P limitation promotes TAG accumulation across diverse microalgal taxa, including *N. oceanica, P. tricornutum, Scenedesmus* sp., and *Chlorella* species, although typically to a lesser extent than under severe N limitation (Mühlroth et al. 2017, Huang et al. 2019, Solovchenko et al. 2019, Hulatt et al. 2020). As summarized in Table 1, this increase in lipid content is often accompanied by changes in FAs composition, most commonly an enrichment in C16 and C18 species and, in some cases, a higher proportion of saturated species. However, compared with N stress, the effects of P limitation on FAs desaturation are less consistent. In some species, desaturation levels remain stable or even increase, whereas in others TAG accumulation is associated with a shift toward more saturated and monounsaturated FAs. These contrasting patterns may reflect differences in metabolic states, species-specific regulations, or experimental conditions.

Importantly, lipid accumulation under P limitation does not always correlate directly with the expression of TAG biosynthetic genes. For example, *Chlorella sorokiniana* shows transient TAG accumulation during early P starvation despite the downregulation of *DGAT* and *LPAT*, followed by a decline in TAG content at later stages (Jin et al. 2021). Similarly, *C. vulgaris* accumulates lipids enriched in saturated FAs under combined CO₂ enrichment and nutrient (N and P) limitation despite reduced *DGAT* expression (Kumari et al. 2021). Together, these observations suggest that TAG accumulation under nutrient stress is rather driven by metabolic carbon flux redistribution and membrane lipid turnover rather than by transcriptional regulation alone.

### Light intensity

Light is the primary energy source sustaining photosynthetic carbon assimilation and therefore fundamentally shapes microalgal metabolism (Yamaoka et al. 2025). In natural aquatic environments, irradiance fluctuates due to turbulent mixing and water movement, exposing microalgae to rapidly changing light regimes. To cope with this variability, microalgae have evolved dynamic photoprotective and metabolic adjustment mechanisms. Besides its central role in photosynthesis, light intensity is one of the key environmental factors influencing TAG productivity (Kwan et al. 2021, Lau et al. 2022). In this section and in Table 2, we summarize the recent research on how high light stress affects both the expression of TAG biosynthetic genes and the lipid content and composition across diverse microalgal species.

**Table 2:** Overview of recent studies describing transcriptional regulation of the key genes encoding TAG-synthesizing enzymes and associated lipid remodeling in microalgae exposed to high light and related light-driven stress conditions.

Species	Clade	Ecology/Trophic mode	Stress type	Stress details	Gene response (Kennedy pathway)	Lipid outcome	FA profile changes	Reference
*Chlamydomonas reinhardtii*	Green algae	Freshwater/Photoautotroph	High light	—	*DGAT3* ▲; *DGAT1* ▲; *PDAT*	TAG ↑	—	Carro et al. (2022)
*Chlamydomonas reinhardtii*	Green algae	Freshwater/Photoautotroph	High light	pgr5 mutant	*DGAT2A* ▲; *PDAT1* ▲	TAG ↑ (60%–70% TL)	—	Chouhan et al. (2022)
*Chlorella zofingiensis*	Green algae	Freshwater/Photoautotroph	High light + salinity	Combined stress	*DGAT1A* ▲; *DGTT5* ▲; *PDAT* ▲	TAG ↑ (~15-fold); TFA ↑	PUFA ↓	Kou et al. (2020)
*Fragilariopsis cylindrus*	Diatom	Marine/Photoautotroph	Light–dark transition	Diurnal cycle	*GPAT* ▲; *LPAAT* ▲; *DGAT* ▲ (light phase)	TAG ↑ (light); ↓ (dark)	PUFA stable	Joli et al. (2024)
*Haematococcus pluvialis*	Green algae	Freshwater/Photoautotroph	High light	Short-term (24 h)	*DGAT* ▲	TAG ↑	MUFA ↑; SFA ↑; PUFA ↓	Scodelaro Bilbao et al. (2020)
*Haematococcus pluvialis*	Green algae	Freshwater/Photoautotroph	High light	Time-dependent	*DGAT1* ▲; *DGTT1–4* ▲	TAG ↑; carotenoid esters ↑	—	Ma et al. (2022)
*Phaeodactylum tricornutum*	Diatom	Marine/Photoautotroph	High light	—	*GPAT* ▲; *LPAAT* ▲; *PAP* ▲; *DGAT1A* ▲; *DGAT2B* ▲; *DGAT2D* ▲; *PDAT* ▲	TL ↑ (~50%); TAG ↑ (~18×)	SFA, MUFA ↑; PUFA ↓	Ding et al. (2023)
*Tetradesmus obliquus* (WT, slm1)	Green algae	Freshwater/Photoautotroph	Light–dark + N limitation	Diurnal + combined stress	*DGAT* ▲ (dark phase)	TAG stable (WT); ↑ (slm1)	—	León-Saiki et al. (2020)

Taxa are organized according to major phylogenetic groups, with information on ecological origin and trophic strategy provided. Changes in transcript levels are denoted using arrowheads (▲, increased expression; ▼, decreased expression; no symbol, no change in expression.). Alterations in lipid content and FA composition are indicated using standard arrows, where ↑ indicates increase and ↓, decrease. Variations in experimental setups (e.g. high irradiance, light–dark cycles, or combined stress treatments) are specified.

Similar to nutrient stress, changes in light conditions, particularly high light exposure, also trigger substantial, yet species-specific, changes in the expression of genes involved in TAG biosynthesis. Studies in the model diatom *P. tricornutum* provide clear examples of how light stress modulates these pathways. High light exposure resulted in the differential regulation of key TAG biosynthetic genes (Ding et al. 2023). Specifically, a plastid-localized *GPAT* isoform was upregulated, whereas its extraplastidic counterpart remained largely unchanged, indicating possible compartment-specific control of acyl flux. Similarly, both *PtPAP1* and *PtPAP2* were sequentially upregulated, suggesting the coordinated activation of downstream steps in TAG assembly. Such culture conditions resulted also in the isoform-specific upregulation of DGAT-encoding genes, including *DGAT1A, DGAT2B*, and *DGAT2D* (Ding et al. 2023, Table 2). This isoform-specific regulation of *DGAT* genes under high light appears to be a more common feature across diverse microalgal taxa. In the model alga *C. reinhardtii*, only *CrDGAT2A* was significantly induced under high light, despite the presence of multiple *DGAT* isoforms, and this was accompanied by increased expression of *PDAT*, suggesting the involvement of both acyl-CoA–dependent and acyl-CoA–independent pathways in TAG accumulation (Chouhan et al. 2022). Similarly, in *Chromochloris zofingiensis*, only *CzDGAT1A* and *CzDGTT5* were upregulated under elevated light intensity (Kou et al. 2020). A comparable response was observed in also *H. pluvialis*, where high light strongly induced multiple DGAT isoforms, particularly during the early phase of exposure (Ma et al. 2022).

Interesting observations also come from studies on the temporal regulation of genes involved in TAG biosynthesis under light–dark cycles. Transitions between light and dark conditions can dynamically modulate gene expression, as demonstrated in the diatom *Fragilariopsis cylindrus*, where *GPAT, LPAAT*, and *DGAT* expression was induced during the light phase (Table 2). In contrast, in *Tetradesmus obliquus, DGAT* expression peaked during the dark phase of the cycle, suggesting that TAG biosynthesis is under diurnal control (León-Saiki et al. 2020).

In addition to temporal regulation, light-dependent TAG synthesis may also involve alternative pathways for TAG synthesis. In *C. reinhardtii*, the expression of the soluble *DGAT3* isoform increased markedly following a transition from low to high light, suggesting a potential contribution of this specific plastid-associated TAG biosynthesis pathway to overall lipid accumulation under high light intensities (Carro et al. 2022).

As mentioned above, high light exposure generally promotes substantial TAG accumulation accompanied by the coordinated remodeling of FAs composition. In many microalgal species, this response very often involves changes in the acyl pool toward saturated and monounsaturated FAs, often at the expense of polyunsaturated species (Table 2). For example, in *P. tricornutum* grown under high light conditions, a ~50% increase in total lipid content and an approximately 18-fold rise in TAG levels were observed, together with an enrichment in C14:0, C16:0, and C16:1 and a decline in PUFAs such as C16:2, C16:3, C18:3, and C20:5 (Ding et al. 2023). Comparable patterns have been reported in several other taxa. In *C. reinhardtii*, high light induced strong TAG accumulation, particularly in photoprotective mutants such as *pgr5* with a disrupted Proton Gradient Regulation 5-encoding gene, where TAG levels reached up to 60%–70% of total cellular lipids (Chouhan et al. 2022). Similarly, *N. oceanica* IMET1 exhibited increased lipid content with an enrichment in C16 and C18 FAs (Wang and Jia 2020), while *C. zofingiensis* showed up to a 15-fold increase in TAG accompanied by a strong reduction in PUFAs levels after high light exposure (Kou et al. 2020). A comparable trend has also been observed in *H. pluvialis*, where analogical stress conditions promoted TAG accumulation together with increased proportions of saturated and monounsaturated FAs and reduced PUFAs content, alongside enhanced carotenoid ester formation (Ma et al. 2022). An interesting exception to this general trend was observed in *Scenedesmus acuminatus*, where high light induced TAG accumulation alongside increased PUFA levels, indicating that lipid remodeling under light stress can follow distinct, species-specific metabolic strategies (Zhang et al. 2021).

Overall, high light responses in microalgae show substantial variability in both TAG levels and FAs composition. In contrast to nutrient stress, where lipid remodeling often follows more consistent patterns, light-driven responses seem to be more heterogeneous, likely reflecting differences in redox balance, photoprotective capacity, and metabolic flexibility among diverse microalgal taxa (Maltsev et al. 2021). Consequently, this variability in TAG accumulation under high light appears to result from a dynamic interplay between transcriptional regulation, redox-driven metabolic adjustments, and species-specific physiological strategies.

### Salinity stress

Salinity is an important environmental factor affecting microalgal physiology, particularly in the context of large-scale cultivation strategies aimed at reducing freshwater use. Although it has been less extensively studied than nutrient or light stress, salinity exerts pronounced effects on cellular metabolism, including TAG accumulation and FAs composition (Cañavate and Fernández-Díaz 2022). Elevated salt concentrations impose hyperosmotic stress, disrupting ionic balance, altering cellular turgor, and challenging membrane stability. In response, microalgae undergo a range of adaptive metabolic adjustments, with the activation of TAG biosynthetic pathways emerging as a consistent and widely observed feature across diverse species, as supported by several recent studies in diverse microalgae (Table 3).

**Table 3:** Compilation of recent studies on transcriptional responses of TAG biosynthetic machinery and associated lipid remodeling in microalgae exposed to salinity and osmotic stress conditions.

Species	Clade	Ecology/Trophic mode	Stress type	Stress details	Gene response (Kennedy pathway)	Lipid outcome	FA profile changes	Reference
*Chlorella* sp. DT025	Green algae	Freshwater/Photoautotroph	Salt stress	15–45 g/L NaCl (dose-dependent)	*GPAT* ▲	Lipid content ↑ (up to 65% DW); lipid yield ↑ (optimal at 15–30 g/L), ↓ at 45 g/L	—	He et al. (2024)
*Chlorella* sp. TLD6B	Green algae	Freshwater/Photoautotroph	Salt stress	0.4–0.6 M NaCl	*GPAT* ▲; *LPAAT* ▲; *DGAT* ▲	Lipids ↑ (23.4 → 58.7% DW), ↓ at ≥0.6 M; productivity ↑ (optimal at 0.4 M)	SFA (C16:0) ↑; MUFA (C18:1) ↑; PUFA ↓	H. Li et al. (2021a)
*Chlorella kessleri*	Green algae	Freshwater/Photoautotroph	Combined stress	Osmotic + nutrient limitation	*DGAT2a* ▲; *DGAT2b* ▲	TFA ↑	—	Otaki et al. (2019)
*Chlamydomonas reinhardtii*	Green algae	Freshwater/Photoautotroph	Salt stress	NaCl, KCl, LiCl	*DGTT1* ▼; *DGTT2* ▼	Neutral lipids ↑ (2.0–3.3×)	SFA ↑; PUFA ↓	Atikij et al. (2019)
*Chromochloris zofingiensis*	Green algae	Freshwater/Photoautotroph	Salt stress	—	*DGTT5* ▲; *GPAT2* ▲; *LPAAT* ▲; *PAP3* ▲; *PDAT* ▲	Total FA ↑; TAG ↑	MUFA ↑; PUFA ↓	Mao et al. (2020a)
*Graesiella emersonii* (NC-M1)	Green algae	Freshwater/Photoautotroph	Combined stress	NaCl + glucose	*GPAT* ▲; *DGAT2* ▲; *DGAT3* ▲	Lipid content ↑; productivity ↑; neutral lipids ↑ (~47.6%)	SFA, MUFA ↑; PUFA ↓	Mandal and Chaurasia (2021)
*Schizochytrium* sp. M326	Thraustochytrid	Marine/Heterotroph	High salinity (ALE)	Long-term adaptive evolution	*GPAT* ▲; *DGAT* ▲	Lipid yield ↑ (~50%); productivity ↑ (~196%)	SFA ↑; PUFA ↓	Sun et al. (2018b)
*Scenedesmus* sp. IITRIND2	Green algae	Freshwater/Photoautotroph	High salinity	Artificial seawater	*DGAT* ▲; *PDAT* ▲	Total lipids ↑; TAG ↑	SFA, MUFA ↑; PUFA ↓	Arora et al. (2019)

Species are arranged according to major phylogenetic groups, with ecological origin and trophic mode indicated. Changes in expression profiles are represented by arrowheads (▲, increased expression; ▼, decreased expression). Lipid-related responses are indicated using standard arrows, where ↑ indicates increase, and ↓ decrease in content. Variations in experimental conditions (e.g. salt concentration, acclimation strategies, or combined treatments) are specified.

For example, in *C. zofingiensis*, exposure to elevated NaCl concentrations led to the increased expression of GPAT2- and PAP3-encoding genes, along with the upregulation of *DGTT5* and *PDAT*, suggesting the joint involvement of both these TAG biosynthetic routes (Mao et al. 2020a). Similarly, the coordinated upregulation of multiple Kennedy pathway genes, including *GPAT, LPAAT*, and *DGAT*, has been observed in *Chlorella* species under salt stress (Table 3). Comparable transcriptional responses have also been documented in other taxa, including marine and halotolerant microalgae. In *Schizochytrium* sp. ALE-150, salinity stress resulted in a threefold increase in *GPAT* expression and a 2.9-fold increase in *DGAT* transcript levels (Sun et al. 2018b). The desert-derived strain *Chlorella* sp. DT025 also exhibited progressively increasing *GPAT* expression with rising NaCl concentrations, indicating a dose-dependent transcriptional response (He et al. 2024). A similar trend was observed also in *Scenedesmus* sp. IITRIND2, where acclimation to artificial seawater triggered a 6-fold upregulation of a DGAT-encoding gene (Arora et al. 2019).

As expected from these transcriptional patterns, salinity stress is frequently accompanied by the increased accumulation of lipids, particularly TAG (Table 3). For example, in *C. zofingiensis*, salt exposure resulted in a 3- to 4-fold increase in total lipid content and a 5-fold rise in TAG levels (Mao et al. 2020a). Similarly, in *Schizochytrium* sp. ALE-150, lipid yields increased by 50%, while lipid productivity nearly doubled under salt stress (Sun et al. 2018b). In *Chlorella* sp. DT025, lipid content reached up to 65% of the dry cell weight (He et al. 2024), whereas in *Cyamus kessleri*, combined osmotic and nutrient stress resulted in an increase of the FAs content by nearly 4-fold (from 9.3% to 34.7%) (Otaki et al. 2019). Importantly, lipid accumulation under salinity stress often follows a concentration-dependent pattern, with moderate salinity enhancing lipid synthesis, while excessive salt levels suppress growth and ultimately reduce lipid productivity (Table 3). In diverse microalgal species, this increase in lipid content is usually accompanied by consistent changes in FAs composition, most notably the enrichment in saturated and monounsaturated species such as C16:0, C16:1, and C18:1, along with a reduction in PUFAs. These compositional changes were observed in divergent species such as *Scenedesmus, Chlorella*, and *Schizochytrium* (Sun et al. 2018b, Arora et al. 2019, H. Li et al. 2021a) and indicate rather conserved metabolic response associated with osmotic stress adaptation.

### Temperature shifts

Microalgal metabolism is strongly influenced also by temperature changes, which directly affect enzyme kinetics, membrane fluidity, photosynthetic performance, as well as central carbon pathways (T. Zhao et al. 2020a, Ferrer-Ledo et al. 2023, Abdelkarim et al. 2025). In large-scale outdoor cultivation systems, daily and seasonal temperature fluctuations can therefore have a strong impact on both biomass productivity and lipid yields (Ferrer-Ledo et al. 2023, Abdelkarim et al. 2025). At the cellular level, temperature changes trigger a range of responses, among which coordinated lipid remodeling is one of the most prominent, often leading to enhanced TAG accumulation and characteristic changes in FAs composition. Such a mechanism has been proposed to serve as part of the cellular strategy to maintain metabolic stability under thermal fluctuations (Barten et al. 2022, Yang et al. 2022b, Shang et al. 2024). As shown by several recent studies summarized below and in Table 4, these responses are, in turn, driven by changes in the expression of genes involved in lipid metabolism, including the key enzymes of the Kennedy pathway.

**Table 4:** Recent studies reporting transcriptional regulation of the Kennedy pathway genes and associated changes in lipid metabolism in microalgae under temperature stress.

Species	Clade	Ecology/Trophic mode	Stress type	Stress details	Gene response (Kennedy pathway)	Lipid outcome	FA profile changes	Reference
*Auxenochlorella protothecoides*	Green algae	Freshwater/Photoautotroph	Heat stress	32°C vs. 28°C	*GPAT* ▲; *DGAT* ▲; *PDAT* ▲	Total lipids ↑ (~158%); TAG ↑	—	Yang et al. (2022b)
*Isochrysis galbana*	Haptophyte	Marine/Photoautotroph	Heat stress	35°C	*DGAT* ▲	TAG ↑	—	Cao et al. (2019)
*Dunaliella bardawil*	Green algae	Marine/Photoautotroph	Heat stress	42°C	*DGAT* ▼	TAG ↓ (hydrolysis; DAG redirected to membranes)	PUFA-rich membrane lipids ↑	Liang et al. (2020)
*Phaeodactylum tricornutum*	Diatom	Marine/Photoautotroph	Heat stress	—	*DGAT2* ▼	Neutral lipids ↓	PUFA ↓ in TAG	Feijão et al. (2020)
*Crypthecodinium* sp. SUN	Dinoflagellate	Marine/Heterotroph	Temperature variation	15–35°C vs. 25°C optimum	*DGAT2A–D* ▼; *PDAT* ▼ (at extremes); *GPAT* ▲; *LPAAT* ▲	Total FA ↓ at extremes	MUFA, DHA ↑; SFA ↓	Fu et al. (2023)
*Schizochytrium* sp. HX-308	Thraustochytrid	Marine/Heterotroph	Heat stress	34°C	*DGAT* ▲; *GPAT*; *LPAAT*; *PAP*	TAG ↑	PUFA ↑; SFA/MUFA ↓	Hu et al. (2022)
*Schizochytrium* sp. HX-308	Thraustochytrid	Marine/Heterotroph	Cold stress	15°C	*GPAT* ▲; *LPAAT* ▲; *PAP* ▲; *DGAT* ▲	TAG ↓; lipid remodeling ↑	PUFA ↓; SFA/MUFA ↑	Hu et al. (2022)
*Aurantiochytrium* sp. SZU445	Thraustochytrid	Marine/Heterotroph	Cold stress	5°C	*GPAT* ▲; *LPAAT* ▲; *PAP* ▲	TAG ↑; total lipids ↑	PUFA ↑	Song et al. (2022)

Species are categorized by major phylogenetic groups, with ecological origin and trophic mode indicated. Variations in transcript levels are presented using arrowheads, where ▲ indicates upregulation, ▼ indicates downregulation and no symbol, no change in expression. Lipid-related responses are indicated using ↑ for increases and ↓ for decreases in the given lipid content. Experimental conditions, including heat and cold stress as well as deviations from optimal growth temperature, are specified.

#### Heat stress

Elevated temperatures appear to have more variable effects on gene expression in microalgal cells compared to other stress conditions (Table 4). In some microalgae, heat stress is associated with an evident upregulation of TAG biosynthetic genes. For example, in the marine alga *Isochrysis galbana*, heat stress (35°C) resulted in the increased expression of *DGAT* along with the downregulation of genes encoding TAG lipases, indicating that enhanced TAG synthesis might be coupled with reduced lipid turnover (Cao et al. 2019). Similarly, in *Auxenochlorella protothecoides* subjected to heat stress Yang et al. (2022b) observed the coordinated upregulation of *GPAT, DGAT*, and *PDAT*, indicating the activation of both DGAT- and PDAT-mediated TAG assembly routes. In contrast, other species exhibit the opposite response, with the downregulation of key the Kennedy pathway genes under heat stress. In *Dunaliella bardawil, DGAT* expression was found to decrease at 42°C (Liang et al. 2020), and in *P. tricornutum*, elevated temperature was similarly associated with the reduced expression of *PtDGAT2* (Feijão et al. 2020). Comparable expression patterns have been reported also for the marine dinoflagellate *Crypthecodinium* sp. SUN, where *DGAT* genes were downregulated under temperature extremes (Fu et al. 2023).

With respect to lipid content and composition, the effects of heat stress on microalgal cells are similarly variable to the transcriptional patterns described above, as it can either promote TAG accumulation or induce TAG mobilization depending on the species (Table 4). In *I. galbana* and *A. protothecoides*, elevated temperature treatments resulted in increased TAG accumulation and a higher total lipid content, respectively (Cao et al. 2019, Yang et al. 2022b). In contrast, in *D. bardawil*, accumulated TAGs were proposed to be hydrolyzed into DAG and redirected toward membrane lipid synthesis under extreme heat, supporting a shift from storage to membrane repair (Liang et al. 2020). In turn, in *Crypthecodinium* sp. SUN, lipid content peaked at intermediate temperatures (~25°C), while both lower and higher temperature regimes resulted in a reduction of TFAs levels (Fu et al. 2023). These changes were also accompanied by changes in FAs composition, including the increased accumulation of PUFAs (e.g. DHA) at temperature extremes and higher proportions of saturated FAs under optimal growth conditions. In general, heat stress is frequently associated with the substantial remodeling of the FAs pool, involving changes in the relative proportions of saturated and unsaturated species, although the direction of these shifts varies among species. These patterns suggest that thermal responses in microalgae rely on a dynamic, tightly coordinated balance between TAG storage and membrane lipid remodeling that supports cellular homeostasis.

#### Cold stress

Compared to heat stress, low temperature has also been reported to affect the expression of genes directly governing TAG synthesis in several microalgal species, however, these transcriptional changes often do not necessarily translate into increased TAG accumulation (Table 4). As shown in *Schizochytrium* sp., exposure to 15°C resulted in the coordinated upregulation of all the major Kennedy pathway genes (Hu et al. 2022). Similarly, omics analyses in *Aurantiochytrium* sp. revealed a strong induction of *GPAT, LPAAT*, and *PAP* in response to growth at 5°C (Song et al. 2022). Nevertheless, despite this apparent activation of the TAG biosynthetic machinery in these species, their lipid-level responses remain variable. In *Schizochytrium* sp., cold stress led to reduced TAG content together with the extensive remodeling of membrane lipids, supported by lipidomic and transcriptomic evidence (Hu et al. 2022). In contrast, *Aurantiochytrium* sp. displayed increased TAG and total lipid content under similar conditions (Song et al. 2022), consistent with the transcriptional activation of TAG biosynthesis and emphasizing species-specific regulatory outcomes.

Recent studies further show that cold stress is associated also with characteristic changes in FAs composition, most notably an increased degree of their unsaturation (Table 4). This pattern is particularly evident in polar microalgae such as *Chlamydomonas malina*, where low temperatures (4–8°C) promote enhanced PUFAs production (Schulze et al. 2019, Morales-Sánchez et al. 2020). These changes may reflect the membrane lipid remodeling required to maintain membrane fluidity under low-temperature conditions, however, it cannot be excluded that in other microalgal systems, FAs remodeling may follow different trajectories, given the species-specific metabolic adjustments across microalgal genera.

Noteworthy, some cold-adapted strains exhibit increased lipid accumulation when shifted to moderate temperatures. For example, *Ankistrodesmus, Chlorella*, and *Scenedesmus* strains showed substantial increases in total lipid content in response to a temperature rise from 8 to 25°C (Rezaei et al. 2024), suggesting that lipid responses are influenced more by a deviation from the physiological optimum than by the absolute temperature.

### Other environmental and chemical factors

In addition to the classical abiotic stressors discussed above, microalgae exhibit remarkable metabolic plasticity in response to a wide range of environmental and chemical cues. In diverse microalgal species, it has been shown that factors such as pH shifts, ionizing radiation, ultraviolet (UV) exposure, plant growth regulators, amino acids, or engineered nanomaterials have an effect on TAG biosynthesis and lipid profiles. This section, together with Table 5, summarizes recent findings on how these factors affect the expression of genes encoding the key enzymes of TAG biosynthesis as well as the lipid content and FAs composition of the analyzed strains.

**Table 5:** Non-classical environmental and chemical factors influencing TAG biosynthesis-related genes and lipid remodeling in microalgae.

Species	Clade	Ecology/Trophic mode	Modulator type	Treatment details	Gene response (Kennedy pathway)	Lipid outcome	FA profile changes	Reference
*Chlamydomonas reinhardtii*	Green algae	Freshwater/Photoautotroph	pH stress	pH 7.8 vs. 7.0	*DGTT3* ▲; *DGTT1/2/4/5* ▼	Lipids ↑ (~3×); growth ↑	C16:0, C18:1, C18:2 ↑	Ochoa-Alfaro et al. (2019)
*Chlamydomonas reinhardtii*	Green algae	Freshwater/Photoautotroph	pH stress	pH 8.5 vs. 7.8	*DGTT3* ▼; *DGTT1/2/4*	Lipids ↓	C16:0, C18:1 ↑	Ochoa-Alfaro et al. (2019)
*Chlorella pyrenoidosa*	Green algae	Freshwater/Photoautotroph	pH stress	Acidic pH 5.0	*DGAT1* ▲; *DGAT2* ▲	Lipids ↑; yield ↑	—	Hao et al. (2022)
*Phaeodactylum tricornutum*	Diatom	Marine/Photoautotroph	pH modulation	Intracellular alkalization (~pH 9.5)	*GPAT* ▲; *LPAAT* ▲; *DGAT2* ▲	TAG ↑ (~3×)	Slight PUFA ↑	Liu et al. (2022)
*Chlorella pyrenoidosa*	Green algae	Freshwater/Photoautotroph	UV/γ radiation	Dose-dependent	*GPAT* ▲; *DGAT* ▲ (moderate dose); ▼ (high dose)	Lipids ↑ (moderate); ↓ (high)	—	Singh et al. (2022)
*Chlorella sorokiniana*	Green algae	Freshwater/Photoautotroph	γ radiation	Acute exposure	*DGAT4* ▲; *DGAT5* ▲	Lipids ↑ (~48% DW)	C16:0 ↑; PUFA ↓	Tale et al. (2018)
*Chlorella sorokiniana*	Green algae	Freshwater/Photoautotroph	Ionizing radiation	5 Gy	*LPAAT* ▲; *PAP* ▲; *DGAT* ▲	Lipids ↑	C16:0, C16:1 ↑	Stanić et al. (2024)
*Isochrysis galbana*	Haptophyte	Marine/Photoautotroph	UV radiation	—	*DGAT* ▼	TAG ↓	—	Cao et al. (2019)
*Chlorella pyrenoidosa*	Green algae	Freshwater/Photoautotroph	Amino acid	Alanine supplementation	*DGAT2354* ▲; *DGAT3280* ▲	Lipids ↑ (~20%)	—	Li et al. (2024b)
*Graesiella emersonii* (NC-M1)	Green algae	Freshwater/Photoautotroph	Phytohormones	IAA + kinetin + N limitation	*DGAT* ▲	Lipid yield ↑ (~158%); neutral lipids ↑	—	Mandal et al. (2020)
*Phaeodactylum tricornutum*	Diatom	Marine/Photoautotroph	Phytohormone	Methyl jasmonate (MeJA)	*DGAT2A* ▲	TAG ↑ (~1.5×)	—	Chu et al. (2019)
*Phaeodactylum tricornutum*	Diatom	Marine/Photoautotroph	Phytohormones	Salicylic acid + ABA	*DGAT2A* ▼; *DGAT2D* ▼	TAG ↓	—	Chu et al. (2019)
*Phaeodactylum tricornutum*	Diatom	Marine/Photoautotroph	Growth regulator	Paclobutrazol	*LPAAT* ▲	Neutral lipids ↑	C16:0, C16:1, C18:0, C18:1 ↑; PUFA ↓	Yang et al. (2023)
*Dunaliella tertiolecta*	Green algae	Marine/Photoautotroph	Chemical treatment	TEA	*PDAT* ▲; *DGAT* ▼	Lipids ↑; productivity ↑	—	Chen et al. (2020)
*Crypthecodinium* sp. SUN	Dinoflagellate	Marine/Heterotroph	Autophagy inhibitor	3-methyladenine	*GPAT* ▼; *LPAAT* ▼; *PAP* ▼; *DGAT* ▼	Lipids ↓; FA ↓	—	Li et al. (2024a)
*Chlamydomonas reinhardtii*	Green algae	Freshwater/Photoautotroph	Nanoparticles	Biodegradable iBCA-NPs	*DGAT* ▲; *DGTT* ▲; *PDAT* ▲	TAG ↑ (~5×)	—	Lu et al. (2023)
*Chlorella pyrenoidosa*	Green algae	Freshwater/Photoautotroph	Nanomaterials	Carbon nanotubes	*DGAT* ▲	Lipid droplets ↑; total lipids ↓	SFA/UFA ↓	Zhang et al. (2018)
*Desmodesmus* sp.	Green algae	Freshwater/Photoautotroph	Atmospheric and room temperature plasma (ARTP) mutagenesis	—	*GPAT* ▲; *DGAT* ▲; *PDAT* ▲	TAG ↑; lipids ↑	—	P. Li et al. (2021b)

The table integrates the recent studies employing diverse modulators, including pH variation, radiation, metabolic additives, and engineered materials, and summarizes their effects on the transcription of Kennedy pathway genes and lipid phenotypes across diverse species. Directional changes in gene expression are indicated by arrowheads, where ▲ indicates upregulation, ▼ indicates downregulation and no symbol indicates no changes in expression. Changes in lipid content and FA composition are summarized using arrows, where ↑ indicates an increase and ↓ indicates a decrease. Because these modulators differ widely in their mode of action and experimental implementation, the reported responses should be interpreted in the context of specific treatment conditions.

#### pH

Environmental pH strongly influences microalgal growth, carbon acquisition, nutrient availability, and enzyme activity (Yadav and Singh 2021, Alkhamis et al. 2022, Liu et al. 2022). By altering the distribution of dissolved inorganic carbon species (CO₂, HCO₃^−^, and CO₃^2−^), pH directly affects photosynthetic efficiency and carbon partitioning (Abraham et al. 2023, Fan et al. 2024). Accordingly, pH manipulation has been explored as a promising strategy to enhance lipid productivity in microalgae. In diverse taxa, this effect is reflected in significant changes in TAG accumulation under different pH conditions, which are associated with altered transcriptional profiles of the key genes involved in TAG biosynthesis (Table 5). For example, in *Chlorella pyrenoidosa*, acidic conditions (pH = 5.0) led to a strong upregulation of *DGAT1* and *DGAT2* and were associated with enhanced lipid accumulation and the redistribution of carbon from proteins and carbohydrates toward lipids (Hao et al. 2022). Similarly, the coordinated upregulation of genes involved in carbon fixation, FAs synthesis, and TAG assembly, including *GPAT, LPAAT*, and *DGAT2*, was observed in response to intracellular alkalization in *P. tricornutum* (Liu et al. 2022). Among the five DGTT-encoding genes present in *C. reinhardtii*, moderate alkalization (pH = 7.8) of the growth medium resulted in the increased expression of only *CrDGTT3*, whereas a further elevation to pH = 8.5 reduced its expression, indicating both a dose-dependent and an isoform-specific transcriptional response (Ochoa-Alfaro et al. 2019).

These transcriptional changes were found to be reflected, to varying extents, in corresponding changes in the lipid profiles of the analyzed microalgal strains (Table 5). In the aforementioned study by Ochoa-Alfaro et al. (2019), *C. reinhardtii* exhibited up to a 3-fold increase in lipid content at pH = 7.8, accompanied by a shift in the FAs composition toward C16:0, C18:1, and C18:2. Further alkalization, however, reduced total lipid levels, reinforcing the dose-dependent relationship between gene expression and lipid accumulation. In another study, alkaline stress combined with N limitation in *A. protothecoides* resulted in TAG accumulation reaching up to 81% of total lipids, along with an enrichment in MUFAs favorable for biodiesel applications (Andeden et al. 2021). Similarly, mixed microalgal cultures derived from a natural freshwater–wastewater system and composed of diverse taxa, including *Scenedesmus, Chlorella*, and diatoms, showed enhanced lipid productivity under moderately alkaline conditions (pH = 8), together with FA profiles enriched in saturated species (Alkhamis et al. 2022). Notably, some species, such as *C. kessleri*, tolerate a wide pH range (3–11) while sustaining growth and lipid production across these conditions, although maximal lipid productivity is achieved near a neutral pH, reflecting a strong homeostatic regulation of intracellular conditions and metabolism (X. Song et al. 2023b).

Overall, the findings summarized in this section reveal a pronounced interspecies variability in both the transcriptional and metabolic adjustments of microalgal cells to changes in environmental pH, emphasizing the distinct species-specific regulatory strategies of this response.

#### Radiation

As shown by several recent studies (Table 5), radiation, including gamma (γ) and UV exposure, represents another potent abiotic factor capable of reprogramming cellular lipid metabolism and promoting lipid accumulation in diverse microalgae, primarily through controlled oxidative stress and reactive oxygen species (ROS) signaling (Tale et al. 2018, Abo-State et al. 2019, Singh et al. 2022).

Activation of these signaling pathways is often accompanied by changes in the expression profiles of genes directly involved in TAG biosynthesis, although these responses are highly species- and dose-dependent. In *C. pyrenoidosa*, e.g. exposure to moderate doses of UV-B and γ-irradiation was associated with the increased expression of *GPAT* and *DGAT*, whereas higher doses were accompanied by the reduced expression of these genes, suggesting the suppression of TAG biosynthetic pathways under excessive oxidative stress (Singh et al. 2022). A similar regulatory effect was observed in *C. sorokiniana*, where acute γ-irradiation resulted in the upregulation of some *DGAT* isoforms (*DGAT4* and *DGAT5*), accompanied by rapid lipid accumulation (Tale et al. 2018). In contrast, in *I. galbana*, UV exposure resulted in the downregulation of *DGAT* expression and a corresponding decrease in TAG content, indicating that radiation-induced responses may differ substantially between species (Cao et al. 2019).

Controlled radiation exposure was found to trigger significant changes in lipid content and composition in diverse microalgae. Low-dose γ-irradiation, e.g. resulted in significantly increased lipid content and productivity in species such as *Tetraselmis suecica, Dunaliella tertiolecta, P. tricornutum*, and *N. oceanica*, with optimal stimulation observed at sublethal doses (D.H. Jeong et al. 2017a). In *C. sorokiniana* exposed to acute γ-irradiation, the lipid content also increased and reached up to 48% of the dry weight within 3 days and was accompanied by increased levels of saturated FAs (e.g. C16:0) and a reduced PUFAs content (Tale et al. 2018).

Regarding the UV radiation, moderate exposure has been reported to stimulate lipid accumulation in microalgae, whereas higher UV doses negatively affect both biomass and lipid productivity. For example, transcriptomic and physiological analyses in *C. sorokiniana* showed that controlled UV enhanced lipid accumulation, coinciding with the upregulation of FAs biosynthesis genes and driven, at least in part, by ROS signaling (Singh et al. 2022). In contrast, exposure to higher or prolonged UV-B radiation led to reduced growth, decreased chlorophyll and carbohydrate content, and lower lipid productivity, likely reflecting damage to the photosynthetic machinery and overall cellular metabolism (Kumar et al. 2018).

Overall, the contrasting effects of radiation doses on TAG accumulation may reflect differences in cellular tolerance to oxidative and DNA damage. Lower radiation doses often induce moderate stress responses that stimulate lipid remodeling and TAG accumulation as protective mechanisms, whereas higher doses may exceed cellular repair capacity, leading to metabolic disruption and reduced lipid synthesis. Thus, the observed variability in TAG responses likely correlates with species-specific resistance or sensitivity to radiation stress, as well as differences in antioxidant capacity and stress mitigation pathways.

#### Chemical modulators

A wide range of chemical compounds tested in microalgal cultures, including amino acids, plant growth regulators, synthetic chemicals, and nanomaterials, was also shown to affect TAG biosynthesis pathways through diverse regulatory mechanisms.

Amino acid supplementation represents one such example. For instance, in *C. pyrenoidosa*, supplementation of the growth medium with alanine was associated with the upregulation of DGAT-encoding genes (e.g. *DGAT2354* and *DGAT3280*) and a corresponding ~20% increase in lipid content (Li et al. 2024b). Similar effects have been reported for plant growth regulators, although the responses are strongly dependent on both the species and the compound. For example, the combined application of indole-3-acetic acid (IAA) and kinetin under N limitation increased *DGAT* expression in *G. emersonii*, accompanied by enhanced total lipid accumulation (Mandal et al. 2020).

In *P. tricornutum*, treatment with methyl jasmonate (MeJA) was also associated with the selective upregulation of specific TAG biosynthesis genes, including *PtDGAT2A* (and, to a lesser extent, other putative acyltransferases such as *PtWS/DGAT*), in a time- and concentration-dependent manner, rather than the uniform induction of all *DGAT* isoforms (Chu et al. 2019). This transcriptional response coincided with a moderate increase in TAG productivity, reaching ~ 1.5-fold that of the control under optimal MeJA concentrations. In contrast, treatments with salicylic acid (SA) and abscisic acid (ABA) were generally associated with the downregulation of some *DGAT2* isoforms (*PtDGAT2A* and *PtDGAT2D*), and had no effect on TAG content per biomass, indicating that these phytohormones may promote lipid production primarily through effects on growth rather than the direct stimulation of TAG biosynthesis (Chu et al. 2019).

Interestingly, emerging evidence indicates that nanomaterials can also modulate cellular pathways related to lipid metabolism in microalgae (Chen et al. 2019). For example, the supplementation of *C. reinhardtii* cultures with biodegradable nanoparticles was associated with the increased expression of *DGAT, DGTT*, and *PDAT*, accompanied by substantial increases in TAG accumulation, in some cases up to 5-fold (Lu et al. 2023). A similar effect has been reported for carbon nanotubes in *C. pyrenoidosa*, where elevated *DGAT* expression coincided with enhanced TAG formation and shifts in the FAs composition toward increased MUFAs and reduced PUFAs (Zhang et al. 2018).

Notably, however, the transcriptional responses of core Kennedy pathway genes do not always directly align with overall TAG accumulation patterns, suggesting the involvement of compensatory metabolic routes. For instance, in *D. tertiolecta*, triethylamine (TEA) treatment was associated with increased lipid content despite the downregulation of *DGAT*. This response was accompanied by the upregulation of *PDAT*, pointing to a greater reliance on acyl-CoA–independent mechanisms of TAG synthesis (Chen et al. 2020).

Together, these non-classical modulators reveal an additional dimension of complexity in the regulation of TAG biosynthesis in microalgae, where lipid accumulation arises from the interplay of diverse signaling inputs and metabolic plasticity rather than from a single, conserved regulatory framework.

## Integrative Mechanisms Linking Abiotic Stress to Triacylglycerol Biosynthesis

### Stress signaling pathways in triacylglycerol biosynthesis

**Figure 2:**
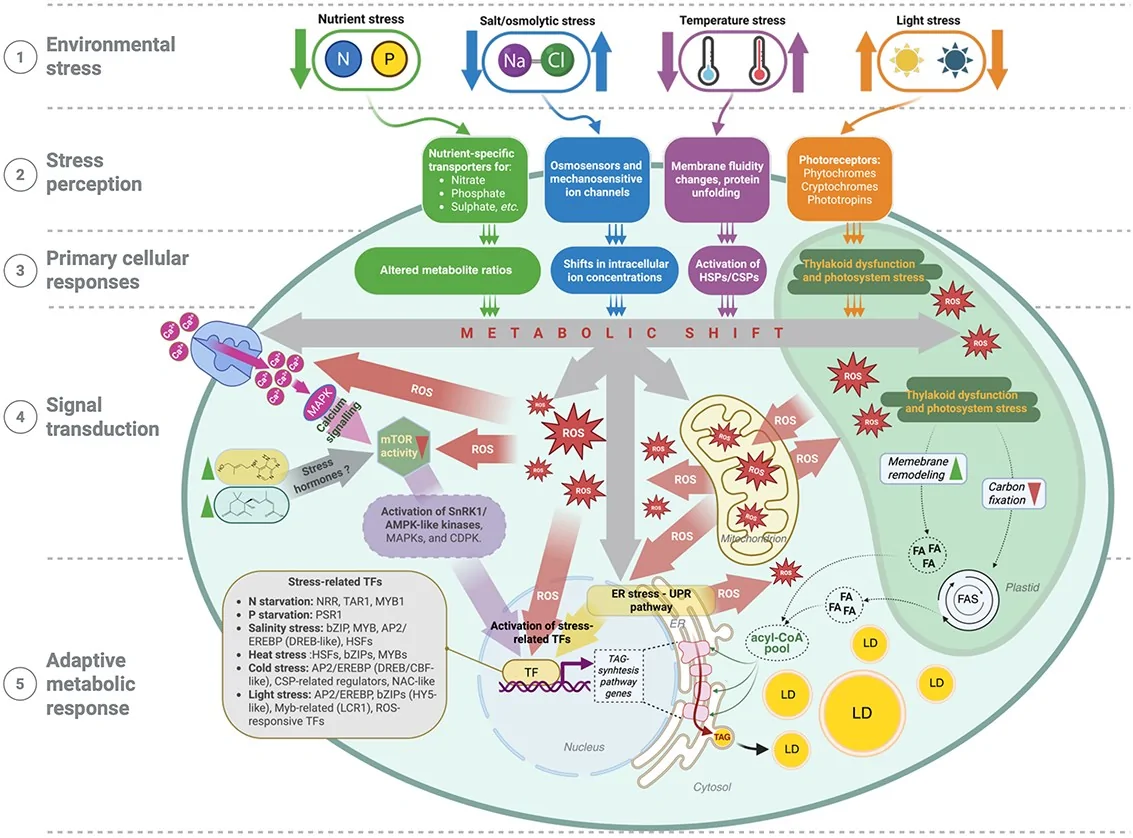
Integrated model of stress perception, signaling, and transcriptional regulation leading to TAG accumulation in microalgae under nutrient limitation, salinity/osmotic stress, temperature fluctuations, and light stress. Environmental cues are detected by nutrient transporters, osmosensors, mechanosensitive ion channels, and photoreceptors, triggering primary cellular responses such as metabolic imbalance, ion flux changes, protein unfolding, and photosynthetic perturbations. These signals converge on the generation of ROS and changes in cytosolic Ca^2+^, which act as central second messengers. ROS and Ca^2+^ activate interconnected signaling pathways, including MAPK cascades, CDPKs, and SnRK1/AMPK-like kinases, while modulating key regulatory hubs such as the TOR pathway and the UPR. Downstream, stress-responsive TFs—including MYB, bZIP, AP2/ERF, and HSF families—coordinate the expression of genes involved in FA synthesis, acyl trafficking, and TAG assembly. The activation of Kennedy pathway enzymes (GPAT, LPAAT, PAP, and DGAT) redirects carbon flux toward TAG biosynthesis, supported by membrane lipid remodeling and metabolite reallocation. The outcome is the accumulation of TAG in LDs, which function in energy storage and protection against stress-induced cellular damage. Created in BioRender. Zienkiewicz, K. (2026). https://BioRender.com/8egg2nl.

Despite the wide diversity of environmental stressors affecting microalgae and the pronounced species-specific variability in their responses, many of these stimuli converge on a relatively conserved set of signaling and metabolic pathways that ultimately promote TAG accumulation. An integrated overview of how diverse abiotic stressors trigger TAG biosynthesis through coordinated sensing, signaling, and transcriptional regulation is presented in Table 6, Supplementary Table S3 and Fig. 2. Within this framework, environmental cues such as nutrient limitation, salinity, temperature fluctuations, and light stress are perceived through distinct yet interconnected sensing systems, including nutrient-specific transporters, osmosensitive ion channels, photoreceptors, and membrane-associated stress sensors (Yamaoka et al. 2018, Mallén-Ponce et al. 2022, Wang et al. 2024). These primary sensing events activate intracellular signaling cascades mediated predominantly by ROS, Ca^2+^ fluxes, and protein kinase networks, which together function as central integrators of stress signals (Fig. 2, Table 6 and Supplementary Table S3). By analogy to other eukaryotic systems, ROS and Ca^2+^ do not act independently in microalgal cells, but instead form a tightly coordinated regulatory network that enables the amplification and diversification of stress responses. Controlled ROS production, particularly in the chloroplasts and mitochondria, serves as a key redox signal that modulates the activity of transcription factors (TFs) and kinase pathways, while Ca^2+^ signaling activates calmodulin-dependent proteins, calcium-dependent protein kinases (CDPKs), and mitogen-activated protein kinases (MAPKs), thereby linking environmental perception to transcriptional reprogramming (Tang et al. 2025; Wang et al. 2024).

**Table 6:** Integrated overview of the regulatory framework linking environmental stress to TAG biosynthesis in microalgae.

Regulatory level	Key components	Representative regulators	Main function	Outcome for TAG biosynthesis
Stress perception	Nutrient, light, osmotic, and ER stress sensors	PII, NIT2; photoreceptors; ion channels; IRE1	Detection of environmental changes and initiation of cellular responses	Activation of stress signaling pathways
Signal integration	Redox and ionic signaling	ROS; Ca^2+^; calmodulin	Integration and amplification of stress signals across cellular compartments	Induction of stress-responsive pathways leading to TAG accumulation
Signal transduction	Kinase and second messenger pathways	MAPKs; CDPKs; cAMP/PKA	Transmission of signals to downstream regulatory networks	Modulation of lipid metabolism and gene expression
Central regulatory hubs	Energy and stress coordination systems	TOR; SnRK1/AMPK; UPR (IRE1–bZIP1)	Coordination of growth, energy status, and stress adaptation	Carbon reallocation from growth to storage (TAG ↑)
Transcriptional regulation	Stress-responsive TFs	MYB; bZIP; AP2/ERF; HSF	Regulation of genes involved in FA synthesis, trafficking, and TAG assembly	Upregulation of GPAT, DGAT, and related enzymes
Metabolic execution	TAG biosynthesis machinery	Kennedy pathway enzymes (GPAT, LPAAT, PAP, DGAT)	Enzymatic conversion of carbon into storage lipids	TAG accumulation and FA remodeling

The table summarizes the main regulatory layers involved in this process, from stress perception and signal integration to transcriptional control and metabolic execution. Representative components and regulators are provided for each level to illustrate how diverse environmental cues are transduced into coordinated cellular responses. Together, these interconnected pathways promote the reprogramming of lipid metabolism and the accumulation of TAG as part of a broader stress adaptation strategy.

As summarized in Fig. 2, these signaling cascades converge on conserved regulatory hubs such as the target of rapamycin (TOR) and SnRK1/AMPK-like kinases, which coordinate the balance between growth and stress adaptation by modulating anabolic and catabolic processes in microalgal cells (Prioretti et al. 2020, Mallén-Ponce et al. 2022). Within this regulatory hierarchy, TOR functions as a master growth regulator under favorable conditions, promoting biosynthetic activity while repressing stress-responsive pathways. Under stress, however, TOR inhibition leads to the activation of the SnRK1/AMPK pathways, the induction of autophagy, and the derepression of stress-responsive TFs, leading to the redirection of cellular metabolism toward energy conservation and storage in the form of TAG (Pérez-Pérez et al. (2017); Prioretti et al. 2020). This regulatory switch represents a key metabolic checkpoint that integrates environmental signals with the cellular energy status (Fig. 2).

Data obtained so far from a wide range of taxonomically diverse microalgal species provide clear evidence that TAG accumulation in microalgae is not merely a passive consequence of a stress-induced metabolic imbalance, but a highly coordinated adaptive response. In this context, the redirection of excess carbon and reducing equivalents into TAG storage contributes to mitigating oxidative stress, preventing the accumulation of cytotoxic intermediates such as FFAs, and maintaining cellular homeostasis. Accordingly, TAG biosynthesis can be viewed as a broadly conserved component of a complex stress-adaptive program that integrates redox balance, energy storage, and membrane remodeling (Fig. 2).

### Transcriptional networks controlling triacylglycerol accumulation

As in other eukaryotic cells, microalgae rely on specific TFs acting downstream of signaling cascades that regulate stress-induced metabolic reprogramming. To date, several microalgal TF families have been identified that directly control genes involved in FAs synthesis, lipid remodeling, and TAG assembly (Table 6 and Supplementary Table S3). Among these, the MYB family TFs have emerged as major regulators of lipid metabolism. For example, an R2R3-MYB protein in *C. reinhardtii* has been shown to directly coordinate the expression of genes involved in FAs trafficking and activation, ensuring an efficient substrate supply for TAG biosynthesis (Choi et al. 2022). Functional analyses in the same model by Zhao et al. (2023) further demonstrated that MYB1 is essential for TAG accumulation, as loss-of-function mutants showed significantly reduced TAG levels, whereas overexpression lines exhibited increased TAG and total FA contents. Importantly, this study also showed that MYB1 regulates the expression of multiple *DGTT* genes, reinforcing its role as a key lipogenic regulator in *C. reinhardtii* (Zhao et al. 2023). A similar role for MYB1 has also been proposed in *C. zofingiensis* by Shi et al. (2022), who showed that MYB1 homologs are transcriptionally induced during N-deprivation-triggered TAG accumulation and coordinate the expression of a broad network of genes involved in *de novo* FAs synthesis, FAs activation and desaturation, membrane lipid remodeling, and TAG assembly.

Basic leucine zipper (bZIP) TFs constitute another major regulatory class linking stress signaling to lipid metabolism. For example, CrbZIP1 from *C. reinhardtii* was shown to be activated under ER stress through unconventional mRNA splicing mediated by CrIRE1, an ER-resident transmembrane sensor (inositol-requiring enzyme 1) with kinase and endoribonuclease activities that detect misfolded proteins and initiate the unfolded protein response (UPR). This pathway plays also a key role in coordinating ER stress responses with lipid remodeling and TAG biosynthesis (Yamaoka et al. 2019, Fig. 2). Similarly, CrbZIP2 was proposed to regulate the balance between membrane lipids and TAG pools under N deprivation, demonstrating well how transcriptional control integrates multiple metabolic pathways (Bai et al. 2021; Table 6 and Supplementary Table S3).

In addition to MYB and bZIP TFs, AP2/ERF family members TFs have also been implicated in TAG regulation across multiple species. For instance, recently NgAP2a from *N. gaditana* has been shown to function as a TF that directly binds to the promoter of the 3-ketoacyl-CoA synthase gene, thus promoting FAs elongation and its overexpression was associated with the increased expression of key lipid biosynthesis genes, including *DGAT*s (Lin et al. 2024). Moreover, in *A. protothecoides* UTEX 2341, a genome-wide analysis identified 18 temperature-responsive TFs, including 6 AP2, 6 ERF, and 6 R2R3-MYB members, under both heat and cold conditions. A co-expression network analysis linked these TFs to multiple lipid metabolism genes, including *GPAT* and *DGAT*, indicating their possible role in the transcriptional regulation of TAG biosynthesis and lipid remodeling under thermal stress (Xing et al. 2021; Table 6 and Supplementary Table S3).

### Molecular endpoints of stress-induced triacylglycerol biosynthesis

While transcriptional networks coordinate the upstream regulation of lipid metabolism under stress, the final execution of TAG biosynthesis ultimately depends on the enzymatic machinery directly responsible for this process (Fig. 2, Table 6, Supplementary Table S3). Among the Kennedy pathway enzymes, DGATs occupy a central position in this process, functioning as key rate-limiting steps through which diverse signaling and transcriptional inputs are translated into TAG accumulation. In this context, the remarkable expansion and diversification of *DGAT* isoforms observed in many microalgal species, particularly *DGTTs*, emerge as a distinctive feature of their metabolic plasticity (Supplementary Table S2). This expansion is unlikely to represent simple genomic redundancy, but, rather, reflects the functional specialization of individual isoforms. This view is supported by their distinct substrate preferences, subcellular localizations, and stress-specific expression patterns reported across diverse microalgae (Liu et al. 2016, Zienkiewicz et al. 2017, Ma et al. 2021). The exceptional diversification of *DGAT*s also provides a mechanistic basis for the ability of microalgae to efficiently channel carbon flux into TAG under a wide range of environmental conditions. This is further supported by the stress-specific regulation of individual *DGAT* isoforms within multigene families. Such patterns are particularly evident in N-deprived *Nannochloropsis* as well as in other species under P limitation, salinity stress, and temperature fluctuations (Yang et al. 2018, Huang et al. 2019, Janssen et al. 2020, Zienkiewicz et al. 2020, J. Song et al. 2023a).

Besides transcriptional control, recent studies also indicate the important role of additional regulatory proteins that influence TAG biosynthesis by modulating upstream metabolic processes and substrate availability. For example, a recent study in *C. reinhardtii* by Li et al. (2025) identified carboxyltransferase interactor 1 (*CrCTI1*) as a negative regulator of ACCase, the rate-limiting enzyme of *de novo* FAs synthesis. The disruption of the CrCTI1-encoding gene resulted in increased FAs production and a substantial increase in TAG accumulation, likely due to enhanced substrate availability for downstream DGAT/DGTT-mediated reactions. Similarly, Hidayati et al. (2019) identified the lipid remodeling regulator 1 (LRL1) as a key transcriptional factor controlling lipid remodeling under P deprivation in the same model species. LRL1 was shown to regulate both the expression of TAG biosynthetic genes and LDs formation, as loss-of-function mutants exhibited reduced *DGTT1* expression and reduced TAG accumulation under P-depleted conditions. Together, these findings demonstrate that the function of DGAT enzymes in microalgae is not only regulated at the transcriptional level but is also tightly linked to the upstream control of FAs supply and metabolic flux.

To sum up, the current evidence presented here further supports a view of TAG biosynthesis as a consequence of integrated cellular regulation, where signaling, gene expression, and metabolic fluxes are continuously aligned with environmental inputs. This systems-level perspective provides a framework for understanding how microalgae balance growth, stress adaptation, and energy storage under fluctuating environmental conditions.

## Biotechnological Implications and Future Perspectives

We believe that this review will serve as a valuable resource for the microalgal research community by providing an up-to-date, integrated perspective on the physiological and genetic mechanisms governing stress-induced TAG biosynthesis, and by identifying the key regulatory principles that can inform future studies and applications. The mechanistic insights discussed above, based on studies across diverse species in microalgal lipid metabolism and stress biology, not only advance our understanding of stress-induced TAG biosynthesis at the physiological and genetic levels, but also provide a framework for developing strategies to enhance lipid production in microalgae. In particular, the identification of regulatory mechanisms linking environmental sensing, intracellular signaling, transcriptional control, and enzymatic execution highlights multiple potential targets for the metabolic engineering of microalgae.

From this perspective, the manipulation of key TFs directly implicated in TAG accumulation in response to abiotic stress represents a promising approach for the coordinated regulation of multiple lipid metabolic pathways, as their activity integrates upstream stress signals with downstream TAG biosynthetic processes. Similarly, targeting central regulatory elements, including TOR and SnRK1, offers additional opportunities to modulate the balance between growth and TAG storage under controlled conditions. Also, targeting specific DGAT variants with distinct substrate preferences or stress responsiveness, together with the modulation of upstream pathways controlling acetyl-CoA supply and FAs synthesis, may further enhance both lipid yield and composition. These approaches clearly illustrate how the regulatory principles governing TAG biosynthesis can be translated into targeted strategies for metabolic optimization. In parallel, physiological insights into stress responses may also help optimize microalgal cultivation strategies. Combinatorial stress conditions, such as nutrient limitation coupled with light modulation, have been shown to enhance TAG accumulation, while emerging approaches involving the chemical or hormonal modulation of signaling pathways may enable more precise control of microalgae-based lipid production. However, these strategies must be carefully balanced against their effects on cellular growth, as conditions that favor TAG accumulation often compromise biomass productivity.

Looking ahead, further progress will depend on a deeper understanding of how stress perception and signal integration operate across diverse microalgal species. Expanding comparative and multi-omics studies will be essential for distinguishing conserved regulatory mechanisms from lineage-specific adaptations, thereby refining both fundamental knowledge and applied strategies. Integrating these insights with systems-level modeling and functional genetics will enable more predictive and efficient approaches to manipulating lipid metabolism.

## Supplementary Material

Bhatnagar_et_al_Supplementary_Tables_pcag059

## Data Availability

No new datasets were generated or analyzed in this study.
