# Supplementary material for: Triacylglycerol synthesis in microalgae under abiotic stress: an updated integrative review
Source: Plant Cell Physiol. 2026 May 7;67(8):1263–86. doi: 10.1093/pcp/pcag059 (PMC13545648; doi:10.1093/pcp/pcag059)
Supplement: Supplementary file 1 [file bhatnagar_et_al_supplementary_tables_pcag059.pdf]

Supplementary Table S1

Taxonomic affiliation, plastid origin, habitat, and trophic characteristics of representative microalgal species discussed in this review. Plastid origin reflects evolutionary acquisition through primary or secondary endosymbiosis.

Species	Major clade	Plastid origin	Habitat	Natural trophic mode	References
Chlamydomonas reinhardtii	Chlorophyta (green algae)	Primary plastid	Freshwater	Photoautotrophic	Merchant *et al.* (2007); Gray (1999)
Chlorella vulgaris	Chlorophyta	Primary plastid	Freshwater	Photoautotrophic	Wakasugi *et al.* (1997); Bhattacharya & Medlin (1995)
Nannochloropsis oceanica	Stramenopile (Eustigmatophyceae)	Secondary plastid (red lineage)	Marine	Photoautotrophic	Wang *et al.* (2014); Vieler *et al.* (2012)
Phaeodactylum tricornutum	Stramenopile (diatom)	Secondary plastid (red lineage)	Marine	Photoautotrophic	Bowler *et al.* (2008); Butler *et al.* (2020)
Isochrysis galbana	Haptophyte	Secondary plastid (red lineage)	Marine	Photoautotrophic	Fang *et al.* (2020); Edvardsen *et al.* (2000)
Crypthecodinium sp.	Dinoflagellate	Secondary plastid	Marine	Heterotrophic	Howe *et al.* (2008); Dauvillée *et al.* (2009)
Dunaliella salina	Chlorophyta	Primary plastid	Hypersaline	Photoautotrophic	Smith *et al.* (2010); Ramos *et al.* (2011)
Haematococcus pluvialis	Chlorophyta	Primary plastid	Freshwater	Photoautotrophic	Shah *et al.* (2016); Luo *et al.* (2019)
Scenedesmus acuminatus	Chlorophyta	Primary plastid	Freshwater	Photoautotrophic	Cho & Lee (2024); Zhang *et al.* (2025)
Cyanidioschyzon merolae	Rhodophyta (red algae)	Primary plastid	Hot acidic springs	Photoautotrophic	Miyagishima & Tanaka (2021)
Aurantiochytrium sp.	Thraustochytrid (Stramenopile)	Non-photosynthetic	Marine	Heterotrophic	Simensen *et al.* (2021); Ganuza *et al.* (2019)

References for Supplementary Table S1:

Bhattacharya, D., Medlin, L. (1995) Phylogeny of plastids: a review based on comparisons of small subunit ribosomal RNA coding regions. *J. Phycol.* 31:489–498.

Bowler, C., Allen, A.E., Badger, J.H. et al. (2008) The Phaeodactylum genome reveals the evolutionary history of diatom genomes. *Nature* 456:239–244.

Butler, T., Kapoore, R.V., Vaidyanathan, S. (2020) Phaeodactylum tricornutum: a diatom cell factory. *Trends Biotechnol.* 38:606–622.

Cho, H.S., Lee, J. (2024) Taxonomic reinvestigation of the genus *Tetradesmus* (Scenedesmaceae; Sphaeropleales) based on morphological characteristics and chloroplast genomes. *Front. Plant Sci.* 15:1303175.

Dauvillée, D., Deschamps, P., Ral, J., Plancke, C., Putaux, J., Devassine, J., Durand-Terrasson, A., Devin, A., Ball, S.G. (2009) Genetic dissection of floridean starch synthesis in the cytosol of the model dinoflagellate Crypthecodinium cohnii. *Proc. Natl. Acad. Sci. USA* 106:21126–21130.

Edvardsen, B., Eikrem, W., Green, J.C., Andersen, R.A., Moon-van der Staay, S.Y., Medlin, L.K. (2000) Phylogenetic reconstructions of the Haptophyta inferred from 18S ribosomal DNA sequences and available morphological data. *Phycologia* 39:19–35.

Fang, J., Lin, A., Yuan, X., Chen, Y., He, W., Huang, J. et al. (2020) The complete chloroplast genome of Isochrysis galbana and comparison with related haptophyte species. *Algal Res.* 50:101989.

Ganuza, E., Yang, S., Amezquita, M., Giraldo-Silva, A., Andersen, R.A. (2019) Genomics, biology and phylogeny of *Aurantiochytrium acetophilum* sp. nov. (Thraustrochytriaceae), including first evidence of sexual reproduction. *Protist* 170:209–232.

Gray, M.W. (1999) Evolution of organellar genomes. *Curr. Opin. Genet. Dev.* 9:678–687.

Howe, C.J., Nisbet, R.E.R., Barbrook, A.C. (2008) The remarkable chloroplast genome of dinoflagellates. *J. Exp. Bot.* 59:1035–1045.

Luo, Q., Bian, C., Tao, M., Huang, Y., Zheng, Y., Lv, Y., Li, J., Wang, C., You, X., Jia, B., Xu, J., Li, J., Li, Z., Shi, Q., Hu, Z. (2019) Genome and transcriptome sequencing of the astaxanthin-producing green microalga *Haematococcus pluvialis*. *Genome Biol. Evol.* 11:166–173.

Merchant, S.S., Prochnik, S.E., Vallon, O. et al. (2007) The Chlamydomonas genome reveals the evolution of key animal and plant functions. *Science* 318:245–250.

Miyagishima, S.Y., Tanaka, K. (2021) The unicellular red alga *Cyanidioschyzon merolae*—the simplest model of a photosynthetic eukaryote. *Plant Cell Physiol.* 62:926–941.

Ramos, A.A., Polle, J., Tran, D., Cushman, J.C., Jin, E., Varela, J.C. (2011) The unicellular green alga *Dunaliella salina* Teod. as a model for abiotic stress tolerance: genetic advances and future perspectives. *Algae* 26:3.

Shah, M.M.R., Liang, Y., Cheng, J.J., Daroch, M. (2016) Astaxanthin-producing green microalga Haematococcus pluvialis: from single cell to high value commercial products. *Front. Plant Sci.* 7:531.

Simensen, V., Voigt, A., Almaas, E. (2021) High-quality genome-scale metabolic model of *Aurantiochytrium* sp. T66. *Biotechnol. Bioeng.* 118:2105–2117.

Smith, D.R., Lee, R.W., Cushman, J.C. et al. (2010) The Dunaliella salina organelle genomes: large sequences, inflated with intronic and intergenic DNA. *BMC Plant Biol.* 10:83.

Vieler, A., Wu, G., Tsai, C.H. et al. (2012) Genome, functional gene annotation, and nuclear transformation of the heterokont oleaginous alga Nannochloropsis oceanica CCMP1779. *PLoS Genet.* 8:e1003064.

Wakasugi, T., Nagai, T., Kapoor, M. et al. (1997) Complete nucleotide sequence of the chloroplast genome from a red alga. *Proc. Natl. Acad. Sci. USA* 94:5967–5972.

Wang, D., Ning, K., Li, J. et al. (2014) Nannochloropsis genomes reveal evolution of microalgal oleaginous traits. *PLoS Genet.* 10:e1004094.

Zhang, Y., Huang, Z., Zheng, H., Wang, Q., Li, A. (2020) Growth, biochemical composition and photosynthetic performance of *Scenedesmus acuminatus* under different initial sulfur supplies. *Algal Res.* 45:101728.

Zhang, M., Zhao, W., Gui, Z. et al. (2025) Advances in lipid production and metabolic engineering in microalgae. *Algal Res.* 104303.

Supplementary Table S2.

Reported copy numbers of DGAT isoforms across representative microalgal taxa. The numbers reflect genome annotations reported in the cited studies and may vary between strains, as marked with asterisks.

Species	DGAT1 copies	DGAT2 (DGTT) copies	DGAT3 copies	Total DGAT genes	Reference
Chlamydomonas reinhardtii	1	5	1	7	La Russa *et al.* (2012); Boyle *et al.* (2012); Bagnato *et al.* (2017)
Nannochloropsis oceanica	1	11-12*	N/A	12-13	Vieler *et al.* (2012); Li *et al.* (2014)
Nannochloropsis gaditana	1	11–12*	N/A	12–13	Wang *et al.* (2014)
Phaeodactylum tricornutum	1	4	N/A	5	Guihéneuf *et al.* (2011); Gong *et al.* (2013)
Chromochloris zofingiensis	2	8	N/A	10	Mao *et al.* (2019)
Coccomyxa sp.	1	4-7*	1	8	Oyama *et al.* (2022); Bagnato *et al.* (2017)
Aurantiochytrium sp.	N/A	4-7*	N/A	4–7	Lan *et al.* (2021); Heggeset *et al.* (2019)
Xanthonema hormidioides	N/A	1	N/A	2	Gao *et al.* (2023)

References for Table S2:

Bagnato, C., Prados, M.B., Franchini, G.R., Scaglia, N., Miranda, S.E. and Beligni, M.V. (2017) Analysis of triglyceride synthesis unveils a green algal soluble diacylglycerol acyltransferase and provides clues to potential enzymatic components of the chloroplast pathway. *BMC Genomics*, 18, 223. https://doi.org/10.1186/s12864-017-3602-0

Boyle, N.R., Page, M.D., Liu, B. et al. (2012) Three acyltransferases and nitrogen-responsive regulator are implicated in nitrogen starvation-induced triacylglycerol accumulation in *Chlamydomonas*. *Journal of Biological Chemistry*, 287, 15811–15825.

Gao, B., Hong, J., Chen, J., Zhang, H., Hu, R. and Zhang, C. (2023) The growth, lipid accumulation and adaptation mechanism in response to variation of temperature and nitrogen supply in psychrotrophic filamentous microalga *Xanthonema hormidioides* (Xanthophyceae). *Biotechnology for Biofuels and Bioproducts*, 16, 12.

Gong, Y., Zhang, J., Guo, X., Wan, X., Liang, Z., Hu, C.J. and Jiang, M. (2013) Identification and characterization of PtDGAT2B, an acyltransferase of the DGAT2 acyl-Coenzyme A:diacylglycerol acyltransferase family in the diatom *Phaeodactylum tricornutum*. *FEBS Letters*, 587(5), 481–487.

Guihéneuf, F., Leu, S., Zarka, A., Khozin-Goldberg, I., Khalilov, I. and Boussiba, S. (2011) Cloning and molecular characterization of a novel acyl-CoA:diacylglycerol acyltransferase 1-like gene (PtDGAT1) from the diatom *Phaeodactylum tricornutum*. *FEBS Journal*, 278, 3651–3666.

Lan, C., Wang, S., Zhang, H. et al. (2021) Cocktail biosynthesis of triacylglycerol by rational modulation of diacylglycerol acyltransferases in industrial oleaginous *Aurantiochytrium*. *Biotechnology for Biofuels*, 14, 246.

La Russa, M., Bogen, C., Uhmeyer, A., Doebbe, A., Filippone, E., Kruse, O. and Mussgnug, J.H. (2012) Functional analysis of three type-2 DGAT homologue genes for triacylglycerol production in the green microalga *Chlamydomonas reinhardtii*. *Journal of Biotechnology*, 162, 13–20.

Li, J., Han, D., Wang, D., Ning, K., Jia, J., Wei, L. et al. (2014) Choreography of transcriptomes and lipidomes of *Nannochloropsis* reveals the mechanisms of oil synthesis in microalgae. *The Plant Cell*, 26(4), 1645–1665.

Oyama, K., Matsuwaki, I., Ito, M., Iwahori, R., Nagata, H., Nakamura, I. et al. (2022) Limited fatty-acid supply from the plastid and active catabolism of triacylglycerol prevent the accumulation of triacylglycerol in *Coccomyxa* sp. strain Obi grown under nitrogen-replete conditions. *Algal Research*, 62, 102620.

Roth, M.S., Cokus, S.J., Gallaher, S.D., Walter, A., Lopez, D., Erickson, E. et al. (2017) Chromosome-level genome assembly and transcriptome of the green alga *Chromochloris zofingiensis* illuminates astaxanthin production. *Proceedings of the National Academy of Sciences of the USA*, 114, 4296–4305.

Vieler, A., Wu, G., Tsai, C.H., Bullard, B., Cornish, A.J., Harvey, C. et al. (2012) Genome, functional gene annotation, and nuclear transformation of the heterokont oleaginous alga *Nannochloropsis oceanica* CCMP1779. *PLoS Genetics*, 8(11), e1003064.

Wang, D., Ning, K., Li, J., Hu, J., Han, D., Wang, H. et al. (2014) *Nannochloropsis* genomes reveal evolution of microalgal oleaginous traits. *PLoS Genetics*, 10, e1004094.

Supplementary Table S3.

This table provides a comprehensive overview of the regulatory components involved in stress-induced triacylglycerol (TAG) biosynthesis in microalgae, integrating evidence from diverse studies across multiple species and stress conditions. Regulatory elements are organized according to their position within the signaling hierarchy, including stress sensing, signal transduction, central regulatory hubs, transcriptional regulators, metabolic control, and final enzymatic steps. For each component or pathway, representative regulators are listed together with their associated stress context, functional role, and experimentally supported effects on Kennedy pathway gene expression and lipid metabolism. Where available, specific examples illustrate how these regulators influence key enzymes such as GPAT, LPAAT, PAP, and DGAT, as well as overall TAG accumulation and fatty acid remodeling. Because many of these regulatory mechanisms are highly context-dependent and may vary across species, experimental conditions, and stress types, the reported effects should be interpreted within their original biological framework. This table is intended as a detailed reference resource complementing the simplified overview presented in Table 6, enabling deeper exploration of the molecular networks underlying TAG biosynthesis in microalgae. Upward arrows (↑) indicate an increase or activation of the described process, pathway, or metabolic output, whereas downward arrows (↓) indicate a decrease or repression. When used in the context of gene expression, arrows reflect changes in transcript abundance of the indicated components; when applied to metabolic outcomes, they denote changes in lipid accumulation or flux through the Kennedy pathway. Bidirectional responses (↑/↓) indicate context-dependent or species-specific effects. References cited in this table correspond to those listed in the main manuscript reference list.

Regulatory level	Component / pathway	Representative regulators	Stress context	Functional role in TAG regulation	Effect on Kennedy pathway / lipids	Key references
Stress sensing	Nutrient sensing	PII proteins; NIT2, PSR1	Nutrient deprivation	Sensing cellular C/macronutrient status and activating nutrient stress-responsive transcriptional programs	Indirect activation of DGAT/DGTT; TAG accumulation ↑	Camargo *et al.* (2007); Bajhaiya *et al.* (2015); Vlasova *et al.* (2023)
Stress sensing	Light sensing	Photoreceptors (phototropins, cryptochromes, rhodopsins, phytochromes), PSII-related stress-induced proteins	High light	Detection of excess irradiance and initiation of redox signaling	Lipid remodeling ↑; TAG ↑	Jaubert *et al.* (2017), Shankar et al. (2022); Redekop *et al.* (2022)
Stress sensing	Osmosensing	Mechanosensitive ion channels; osmosensors	Salinity / osmotic stress	Detection of ionic imbalance and turgor changes; triggers Ca^{2+} influx	Activation of downstream lipid regulatory pathways; TAG ↑	Kaur *et al.* (2022); Shetty *et al.* (2019)
Stress sensing	Temperature variations	Heat shock factors (HSFs), heat shock proteins (HSPs)	Heat stress/cold stress	Heat stress: detection of membrane fluidization leading to protein unfolding and ROS-signaling activation<br>Cold stress: detection of membrane rigidification, triggers Ca^{2+} and ROS signaling	Activation of downstream lipid regulatory pathways; TAG ↑	Barati *et al.* (2019); Hu et al. (2020); Song *et al.* (2023a); Ermilova (2020); Xing *et al.* (2022); Huang *et al.* (2025)
Stress sensing	ER stress sensing	IRE1 (CrIRE1)	ER stress	Detection of misfolded proteins and activation of unfolded protein response (UPR)	DGAT activation; lipid remodeling ↑	Yamaoka *et al.* (2019)

Signal transduction	ROS signaling	Reactive oxygen species (chloroplast, mitochondria)	All stresses	Central redox signaling controlling TFs and kinases	Lipogenic gene expression ↑; TAG ↑	Wang *et al.* (2024)
Signal transduction	Ca^{2+} signaling	Ca^{2+}; calmodulin; CDPKs	All stresses	Secondary messenger linking stress perception to transcription	GPAT/DGAT activation; lipid accumulation ↑	Wang *et al.* (2024)
Signal transduction	MAPK cascades	RAF14; RAF79; MAPKK1; MAPK3; MAPK8	Nutrient, salt, oxidative stress	Signal integration via phosphorylation cascades	Context-dependent lipid regulation; TAG ↑/↓	Yang et al. (2018); Gomez-Osuna *et al.* (2020); Qiao *et al.* (2021)
Signal transduction	cAMP signaling	cAMP; PKA	Nutrient, salinity stress	Regulation of carbon flux and ACCase activity	FA synthesis ↑; TAG ↑	Choi *et al.* (2015); Ding *et al.* (2021)
Regulatory hubs	TOR signaling	TOR kinase	Favorable vs stress conditions	Master regulator balancing growth and stress	TOR inhibition → TAG accumulation ↑	Prioretti *et al.* (2020)
Regulatory hubs	Energy sensing	SnRK1 / AMPK-like kinases	Stress conditions	Activation of catabolic pathways and energy conservation	Carbon redirection toward TAG ↑	Pérez-Pérez *et al.* (2017)
Regulatory hubs	UPR pathway	IRE1–bZIP1 axis	ER stress	Coordination of ER stress response and lipid metabolism	DGAT induction; TAG accumulation ↑	Yamaoka *et al.* (2019)
Transcriptional regulators	MYB family	MYB1 (CrMYB1, CzMYB1, NRR1)	Nutrient stress, general	Regulation of FA trafficking and TAG biosynthesis genes	DGAT/PDAT expression ↑; TAG ↑	López García de Lomana *et al.* (2015); Shi *et al.* (2022); Choi *et al.* (2022); Zhao *et al.* (2023)
Transcriptional regulators	bZIP family	CrbZIP1; CrbZIP2	ER stress; N stress; salinity	Integration of stress signaling with lipid metabolism	Lipid remodeling ↑; TAG ↑	Ji et al. (2018); Yamaoka *et al.* (2019); Bai *et al.* (2021)
Transcriptional regulators	AP2/ERF family	NgAP2a; AP2/ERFs	Temperature; general stress	Regulation of FA elongation and TAG biosynthesis	GPAT/DGAT expression ↑; TAG ↑	Lin *et al.* (2024); Xing *et al.* (2021)
Transcriptional regulators	HSF family	PtHSF1	Heat stress	Heat-responsive transcriptional activation of lipid genes	GPAT/DGAT activation; TAG ↑	Song *et al.* (2023a)
Metabolic regulators	FA synthesis control	CTI1	General metabolic regulation	Negative regulator of ACCase; controls FA supply	FA availability ↑ → TAG ↑	Li *et al.* (2025)
Metabolic regulators	Lipid remodeling regulator	LRL1	Phosphorus deprivation	Coordination of lipid remodeling and TAG synthesis	DGTT1 regulation; TAG ↑	Hidayati *et al.* (2019)
Final executors	TAG synthesis enzymes	DGAT / DGTT isoforms	All stresses	Rate-limiting step in TAG biosynthesis	Direct TAG formation; isoform-specific responses	Multiple references

References for Supplementary Table S3:

Bajhaiya, A.K., Dean, A.P., Zeef, L.A., Webster, R.E., Pittman, J.K. (2016) PSR1 is a global transcriptional regulator of phosphorus deficiency responses and carbon storage metabolism in Chlamydomonas reinhardtii. *Plant Physiol.* 170:1216–1234.

Camargo, A., Llamas, A., Schnell, R.A., Higuera, J.J., González-Ballester, D., Lefebvre, P.A., Fernández, E., Galván, A. (2007) Nitrate signaling by the regulatory gene NIT2 in Chlamydomonas. *Plant Cell* 19:3491–3503.

Vlasova, V., Lapina, T., Statinov, V., Ermilova, E. (2023) N-acetyl-L-glutamate kinase of Chlamydomonas reinhardtii: in vivo regulation by PII protein and beyond. *Int. J. Mol. Sci.* 24:12873.

Jaubert, M., Bouly, J.P., Ribera d'Alcalà, M., Falciatore, A. (2017) Light sensing and responses in marine microalgae. *Curr. Opin. Plant Biol.* 37:70–77.

Shankar, U., Lenka, S.K., Ackland, M.L., Callahan, D.L. (2022) Review of the structures and functions of algal photoreceptors to optimize bioproduct production with novel bioreactor designs for strain improvement. *Biotechnol. Bioeng.* 119:2031–2045.

Redekop, P., Sanz-Luque, E., Yuan, Y., Villain, G., Petroutsos, D., Grossman, A.R. (2022) Transcriptional regulation of photoprotection in dark-to-light transition-more than just a matter of excess light energy. *Sci. Adv.* 8:eabn1832.

Kaur, M., Saini, K.C., Ojah, H., Sahoo, R., Gupta, K., Kumar, A., Bast, F. (2022) Abiotic stress in algae: response, signaling and transgenic approaches. *J. Appl. Phycol.* 34:1843–1869.

Shetty, P., Gitau, M.M., Maróti, G. (2019) Salinity stress responses and adaptation mechanisms in eukaryotic green microalgae. *Cells* 8:1657.

Barati, B., Gan, S.Y., Lim, P.E. et al. (2019) Green algal molecular responses to temperature stress. *Acta Physiol. Plant.* 41:26.

Hu, S., Ding, Y., Zhu, C. (2020) Sensitivity and responses of chloroplasts to heat stress in plants. *Front. Plant Sci.* 11:375.

Song, J., Zhao, H., Zhang, L., Li, Z., Han, J., Zhou, C., Xu, J., Li, X., Yan, X. (2023a) The heat shock transcription factor PtHSF1 mediates triacylglycerol and fucoxanthin synthesis by regulating the expression of GPAT3 and DXS in Phaeodactylum tricornutum. *Plant Cell Physiol.* 64:622–636.

Ermilova, E. (2020) Cold stress response: an overview in Chlamydomonas. *Front. Plant Sci.* 11:569437.

Xing, Q., Tu, H., Yang, M., Chen, G., Tang, Q., Yi, S. et al. (2022) Evaluation of cold tolerance and gene expression patterns associated with low-temperature stress in giant freshwater prawn Macrobrachium rosenbergii. *Aquacult. Rep.* 24:101172.

Huang, D., Cheng, C.Q., Zhang, H.Y., Huang, Y., Li, S.Y., Huang, Y.T., Huang, X.L., Pei, L.L., Luo, Z., Zou, L.G., Yang, W.D., Zheng, X.F., Li, D.W., Li, H.Y. (2025) Heat shock transcription factor-mediated thermal tolerance and cell size plasticity in marine diatoms. *Nat. Commun.* 16:3404.

Yamaoka, Y., Shin, S., Choi, B.Y., Kim, H., Jang, S., Kajikawa, M., Yamano, T., Kong, F., Légeret, B., Fukuzawa, H., Li-Beisson, Y., Lee, Y. (2019) The bZIP1 transcription factor regulates lipid remodeling and contributes to ER stress management in Chlamydomonas reinhardtii. *Plant Cell* 31:1127–1140.

Wang, L., Shi, Q., Pan, Y., Shi, L., Huang, X. (2024) ROS and Ca2+ signaling involved in important lipid changes of Chlorella pyrenoidosa under nitrogen stress conditions. *Planta* 260:39.

Yang, A., Suh, W.I., Kang, N.K., Lee, B., Chang, Y.K. (2018) MAPK/ERK and JNK pathways regulate lipid synthesis and cell growth of Chlamydomonas reinhardtii under osmotic stress, respectively. *Sci. Rep.* 8:13857.

Gomez-Osuna, A., Calatrava, V., Galvan, A., Fernandez, E., Llamas, A. (2020) Identification of the MAPK cascade and its relationship with nitrogen metabolism in the green alga Chlamydomonas reinhardtii. *Int. J. Mol. Sci.* 21:3417.

Qiao, T., Zhao, Y., Zhong, D.B., Yu, X. (2021) Hydrogen peroxide and salinity stress act synergistically to enhance lipids production in microalga by regulating reactive oxygen species and calcium. *Algal Res.* 53:102017.

Choi, Y.E., Rhee, J.K., Kim, H.S., Ahn, J.W., Hwang, H., Yang, J.W. (2015) Chemical genetics approach reveals importance of cAMP and MAP kinase signaling to lipid and carotenoid biosynthesis in microalgae. *J. Microbiol. Biotechnol.* 25:637–647.

Ding, K., Ji, J., Xie, G., Liu, S., Liao, C., Song, K., Wang, Y.Z. (2021) Lipid biosynthesis mediated by the cyclic adenosine monophosphate (cAMP) signaling pathway in Chlorella pyrenoidosa under salt-induced osmotic stress. *Renew. Energy* 180:222–231.

Prioretti, L., Carriere, F., Field, B., Avilan, L., Montané, M.H., Menand, B., Gontero, B. (2020) Targeting TOR signaling for enhanced lipid productivity in algae. *Biochimie* 169:12–17.

Pérez-Pérez, M.E., Couso, I., Crespo, J.L. (2017) The TOR signaling network in the model unicellular green alga Chlamydomonas reinhardtii. *Biomolecules* 7:54.

Yamaoka, Y., Shin, S., Choi, B.Y., Kim, H., Jang, S., Kajikawa, M., Yamano, T., Kong, F., Légeret, B., Fukuzawa, H., Li-Beisson, Y., Lee, Y. (2019) The bZIP1 transcription factor regulates lipid remodeling and contributes to ER stress management in Chlamydomonas reinhardtii. *Plant Cell* 31:1127–1140.

López García de Lomana, A., Schäuble, S., Valenzuela, J. et al. (2015) Transcriptional program for nitrogen starvation-induced lipid accumulation in Chlamydomonas reinhardtii. *Biotechnol. Biofuels* 8:207.

Shi, M., Yu, L., Shi, J., Liu, J. (2022) A conserved MYB transcription factor is involved in regulating lipid metabolic pathways for oil biosynthesis in green algae. *New Phytol.* 235:576–594.

Choi, B.Y., Shim, D., Kong, F., Auroy, P., Lee, Y., Li-Beisson, Y., Lee, Y., Yamaoka, Y. (2022) The Chlamydomonas transcription factor MYB1 mediates lipid accumulation under nitrogen depletion. *New Phytol.* 235:595–610.

Zhao, J., Ge, Y., Liu, K., Yamaoka, Y., Zhang, D., Chi, Z., Akkaya, M., Kong, F. (2023) Overexpression of a MYB1 transcription factor enhances triacylglycerol and starch accumulation and biomass production in the green microalga Chlamydomonas reinhardtii. *J. Agric. Food Chem.* 71:17833–17841.

Ji, C., Mao, X., Hao, J., Wang, X., Xue, J., Cui, H., Li, R. (2018) Analysis of bZIP transcription factor family and their expressions under salt stress in Chlamydomonas reinhardtii. *Int. J. Mol. Sci.* 19:2800.

Bai, F., Zhang, Y., Liu, J. (2021) A bZIP transcription factor is involved in regulating lipid and pigment metabolisms in the green alga Chlamydomonas reinhardtii. *Algal Res.* 59:102450.

Lin, Y., Li, Y., Wu, X., Xu, W., Zhang, Z., Zhu, H., Zhou, H. (2024) NgAP2a targets KCS gene to promote lipid accumulation in Nannochloropsis gaditana. *Int. J. Mol. Sci.* 25:10305.

Xing, G., Li, J., Li, W., Lam, S.M., Yuan, H., Shui, G., Yang, J. (2021) AP2/ERF and R2R3-MYB family transcription factors: potential associations between temperature stress and lipid metabolism in Auxenochlorella protothecoides. *Biotechnol. Biofuels* 14:22.

Song, J., Zhao, H., Zhang, L., Li, Z., Han, J., Zhou, C., Xu, J., Li, X., Yan, X. (2023a) The heat shock transcription factor PtHSF1 mediates triacylglycerol and fucoxanthin synthesis by regulating the expression of GPAT3 and DXS in Phaeodactylum tricornutum. *Plant Cell Physiol.* 64:622–636.

Li, Z., Kim, M., da Silva Nascimento, J.R., Legeret, B., Jorge, G.L., Bertrand, M., Beisson, F., Thelen, J.J., Li-Beisson, Y. (2025) Knocking out the carboxyltransferase interactor 1 (CTI1) in Chlamydomonas boosted oil content by fivefold without affecting cell growth. *Plant Biotechnol. J.* 23:1230–1242.

Hidayati, N.A., Yamada-Oshima, Y., Iwai, M., Yamano, T., Kajikawa, M., Sakurai, N., Suda, K., Sesoko, K., Hori, K., Obayashi, T., Shimojima, M., Fukuzawa, H., Ohta, H. (2019) Lipid remodeling regulator 1 (LRL1) is differently involved in the phosphorus-depletion response from PSR1 in Chlamydomonas reinhardtii. *Plant J.* 100:610–626.
